# A Review of the Antibacterial, Fungicidal and Antiviral Properties of Selenium Nanoparticles

**DOI:** 10.3390/ma16155363

**Published:** 2023-07-30

**Authors:** Dmitry A. Serov, Venera V. Khabatova, Vladimir Vodeneev, Ruibin Li, Sergey V. Gudkov

**Affiliations:** 1 Prokhorov General Physics Institute of the Russian Academy of Sciences, Vavilove St. 38, 119991 Moscow, Russia; dmitriy_serov_91@mail.ru (D.A.S.); venera.khabatova@gmail.com (V.V.K.); 2 Institute of Biology and Biomedicine, Lobachevsky State University of Nizhny Novgorod, Gagarin av. 23, 603105 Nizhny Novgorod, Russia; v.vodeneev@mail.ru; 3 State Key Laboratory of Radiation Medicine and Protection, School for Radiological and Interdisciplinary Sciences (RAD-X), Collaborative Innovation Center of Radiological Medicine of Jiangsu Higher Education Institutions, Suzhou Medical College, Soochow University, Suzhou 215123, China; liruibin@suda.edu.cn

**Keywords:** SeNPs, antibiotic resistance, antimicrobial activity, mechanisms of antibacterial action, cytotoxicity to eukaryotic cells

## Abstract

The resistance of microorganisms to antimicrobial drugs is an important problem worldwide. To solve this problem, active searches for antimicrobial components, approaches and therapies are being carried out. Selenium nanoparticles have high potential for antimicrobial activity. The relevance of their application is indisputable, which can be noted due to the significant increase in publications on the topic over the past decade. This review of research publications aims to provide the reader with up-to-date information on the antimicrobial properties of selenium nanoparticles, including susceptible microorganisms, the mechanisms of action of nanoparticles on bacteria and the effect of nanoparticle properties on their antimicrobial activity. This review describes the most complete information on the antiviral, antibacterial and antifungal effects of selenium nanoparticles.

## 1. Introduction

Despite the high level of medical development, microbial infections remain a significant factor in morbidity and mortality worldwide [1]. Sepsis and septic shock alone account for approximately 30 million clinical cases each year worldwide, of which 6 million are fatal [2]. The development of bacterial resistance to antibiotics is not a new problem and dates back to the 1940s–1960s [3,4]. Resistant microorganisms have a significant impact on human life and economic activity. Antibiotic-resistant bacteria significantly increase the risk of complications and death from bacteremia [1,5].

In addition, antibiotic-resistant bacteria complicate the course of foodborne illness, which accounts for more than a million deaths and 2 billion hospitalizations worldwide over a period of 20 years [6]. Complications of bacterial infections can affect almost all human tissues, organs and systems: gastrointestinal tract (gastritis, stomach ulcer, severe forms of diarrhea), central nervous system (meningitis, encephalitis), kidneys, liver, spleen, musculoskeletal system (reactive arthritis), cardiovascular system (endocarditis) and reproductive system (premature birth, stillbirth) [7,8,9,10,11,12,13]. By 2050, microbial resistance is predicted to cause a decrease in the total population of the Earth by 100–440 million people [14].

Apart from humans, antibiotic-resistant bacteria infect animals and plants, reducing the efficiency of agriculture [15,16,17]. Expected financial losses from antibiotic-resistant bacteria in 2025–2050 could be $85 trillion in GDP and $23 trillion in global trade [18]. The mechanisms of antibiotic resistance include enzymatic modification and inactivation of the antibiotic: hydrolysis, phosphorylation, glycosylation, etc. [19], reduction of cell wall permeability due to lipopolysaccharides or lipid enrichment, and removal of antibiotics from cells using special molecular pumps (multidrug [MDR] efflux pumps) [20].

In addition to bacterial antibiotic resistance, the development of fungal resistance to antifungal drugs is worth noting [21]. More than 300 million severe fungal infections have been registered in the world, of which over a million are fatal [22]. Additionally, the demand for antiviral drugs is increasing, in connection with the resistance of viruses, as well as in connection with the SARS-COV-2 pandemic [23], which affected all countries from 2019 until now. One of the promising ways to solve the problem of microbial resistance is the use of nanotechnology [24,25,26,27]. Antimicrobial nanomaterials operate through disruption of the electron transport chain [28,29], membrane destruction [30], cell division arrest [31], etc., and have garnered considerable attention due to their broad, potent and persistent bactericidal activities.

Unfortunately, recent data indicate the possibility of bacterial resistance to metal NPs and metal oxides [32,33,34,35,36,37,38,39]. Bacterial defense mechanisms include increased flagellin expression for NP sorption as well as pigment release to inactivate metal ions and activation of antioxidant defenses to combat oxidative stress [40,41,42]. Currently, an active search is underway for NPs with antibacterial properties among the nonmetallic chemical elements [33,43,44,45].

**Figure 1:**
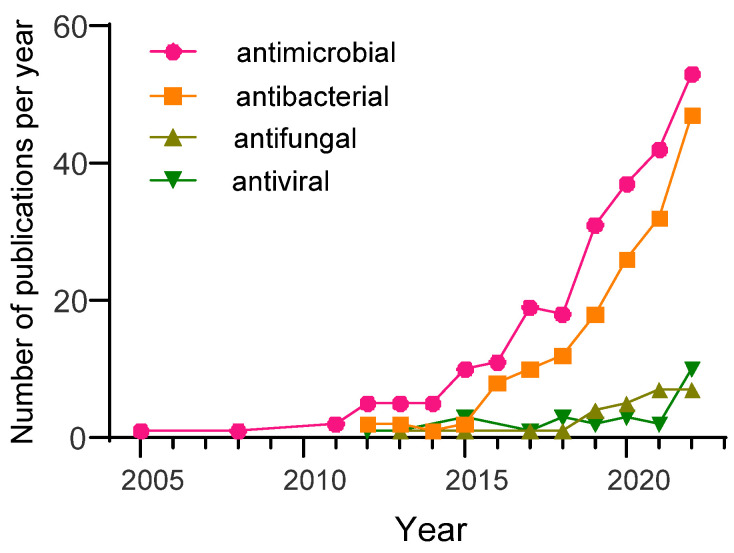
Distribution by year of the publications available in the PubMed database by search keywords ‘selenium nanoparticles antimicrobial activity’ (222 articles); ‘selenium nanoparticles antibacterial activity’ (147 articles); ‘selenium nanoparticles antifungal activity’ (34 articles); ‘selenium nanoparticles antiviral activity’ (25 articles).

Selenium nanoparticles are one such type of nanoparticle. The study of their antimicrobial properties is a young branch of science: the vast majority of research papers (>95%) have been published in the last 10 years. According to PubMed NCBI (https://pubmed.ncbi.nlm.nih.gov/ accessed on 15 May 2023), more than 220 papers have been published with the keywords “selenium nanoparticles antimicrobial activity”, approximately 150 papers with the keywords “selenium nanoparticles antibacterial activity”, approximately 30 papers with the keywords “selenium nanoparticles antifungal activity” and approximately 20 papers with the keywords “selenium nanoparticles antiviral activity” (see Figure 1). It is important to note that every year, an increasing number of researchers pay attention to this problem. Over the past few years, research publication activity worldwide on SeNP antimicrobial properties has been growing by approximately 25–30% per year. Over 100 articles were published in 2022.

The reality of our days is the resistance of microorganisms (bacteria, fungi and viruses) to modern antibiotic, antifungal and antiviral drugs. Today, the scientific community is faced with the task of searching for new potential molecular structures to solve the problem of therapy in bacterial, fungal and viral pathogenesis. Selenium nanoparticles act as such antimicrobial agents. These nanoparticles have a fairly wide range of applications in the biomedical industry. For example, they can affect the activity of neutrophils: this immunomodulatory property of selenium nanoparticles can potentially be used in the treatment of cancer and other diseases associated with inflammation [46]. Moreover, selenium nanoparticles have a cytoprotective effect on the cells of the cerebral cortex under conditions of ischemia/reoxygenation [47]. The mechanism of action of this effect is based on the fact that nanoparticles regulate the expression of protective proteins in the cells of the cerebral cortex and reduce the total level of Ca^2+^ ions. The combination of selenium nanoparticles with the flavonoid taxifolin makes it possible to increase the neuroprotective effect in the cells of the cerebral cortex under conditions of ischemia/reoxygenation [48]. Beyond the immunomodulatory and neuroprotective effects, selenium nanoparticles also demonstrate anticancer effects. Selenium-sorafenib nanocomplexes showed better anticancer effects on HepG2 hepatic carcinoma cells than pure sorafenib [49]

**Figure 2:**
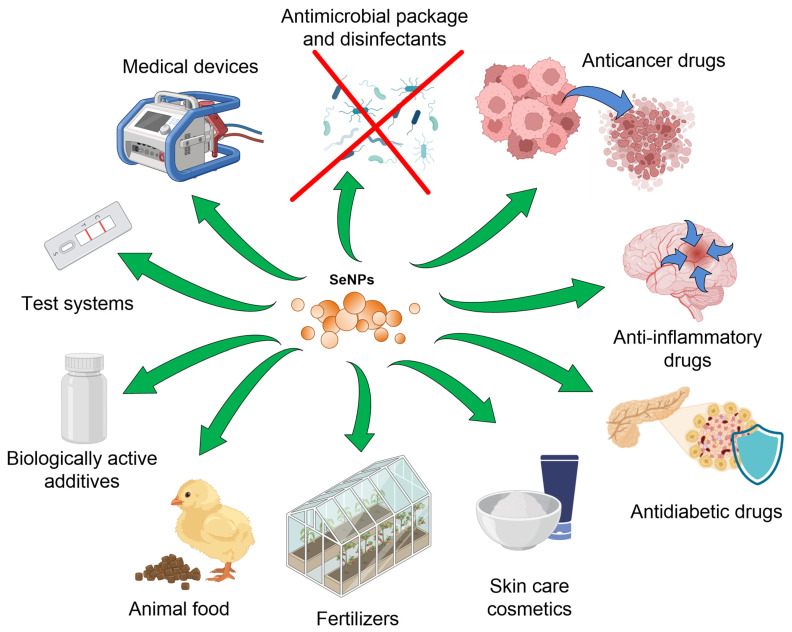
Possible commercial products based on SeNPs (References are given in the text).

According to patent searches and recent publications, SeNPs may have the several potential commercial applications (Figure 2).

The first application is the development of nutritional supplements for humans and veterinary needs [50]. It is noteworthy that SeNPs provide a more dosed supply of Se to the body compared to alternative sources, such as selenium cysteine. In particular, SeNPs, when administered orally to mice, causes less pronounced toxicity (survival is 4.5–5 times higher) and liver failure compared with the same amount of selencysteine also administered orally [51]. SeNPs can also be used in veterinary medicine as immunomodulatory agents. A drug based on nonspecific IgG/SeNPs for the correction of immunization in veterinary use did not have acute toxicity to laboratory mice [52].

The second application is the development of a test system for virus detection, for example, a test strip for the detection of anti-SARS-CoV-2 IgM and IgG in human serum and blood [53,54].

The third application is the creation of antimicrobial coatings for medical devices and personal care products [55,56]. Polyurethane/SeNP and polyvinylchloride/SeNP composites (polymers with NP coatings) inhibited the growth of bacteria *Staphylococcus aureus*, *Staphylococcus epidermidis*, *Pseudomonas aeruginosa*, *Staphylococcus aureus*, *Escherichia coli*, *Streptococcus pneumoniae*, etc., and fungi *Cryptococcus neoformans*, *S. cerevisiae*, *Rhodotorula rubra*, *C. albicans*, etc. [57,58]. The development of dressings and bandaging materials based on SeNPs to accelerate infected wound healing is also possible [59,60,61]

The fourth application is cancer prevention and treatment [62]. Anticancer drugs based on SeNPs cause the generation of ROS, which cause ER stress and the development of mitochondrial dysfunction. These processes enhance apoptosis of tumor cells [51,63]. Typically, to improve anticancer properties, SeNPs are functionalized with polymers or other biological antigens: arabinogalactans against A549 and MCF-7 cells (apoptosis induction), HepG2, polysaccharide–protein complexes (MCF-7 apoptosis induction), Ru(II)thiol/SeNPs (suppress tumor growth and angiogenesis) and polyethylenimine with folic acid [64,65,66]. The accuracy of delivery is ensured by a higher level of expression of folic acid receptors on cancer cells of most forms of cancer compared with normal human cells (for example, HepG2) [67,68]. Hyaluronic acid (A549 cells), ATP (depletion of mitochondrial membrane potential and oxidative stress) and other agents were also described [69,70]. The addition of anticancer agents (sorafenib) to SeNPs significantly increases the activity of others against aggressive cancer lines (increased calcium entry, ER stress and apoptosis of HepG2, gliablastoma A-172) [49,71,72]. Oridoni/SeNP composites have anticancer activity and reduce the viability of RAW264.7, KYSE-150 and EC970 cancer cells by ~50% [73].

The fifth application is the production of cosmeceuticals and nano-cosmeceuticals for skin, hair, nail and lip care and protection from wrinkles, photoaging, hyperpigmentation, dandruff and hair damage [74]. Selenium-containing cosmetics are developed and produced by Riga Stradins University, Phyto-C company (Newark, New York, USA), Cytolnat^®^ (Paris, France), etc.

The sixth application is the production of nanoparticle fertilizers for crop production and soil health. Trichoderma-harzianum-culture-biosynthesized SeNPs (60 nm) reduced Fusarium beadium and Alternaria albicans growth, and fumonisin and Alternaria toxin production, in wheat crops [75]. The addition of selenium or its compounds (usually selenites, selenates, Se-methlyselenocysteine-containing peptides, etc.) to the soil is a common practice in agriculture [76]. All of these compounds are easily soluble and can quickly be washed out of the soil; no more than 20% of the initial selenium is retained for the next farming cycle [77]. The use of SeNPs should provide a longer and dosed supply of Se to the soil. It has been shown that the addition of 1 μg/L SeNPs to the soil improves seed germination and accelerates the growth of the radish *Raphanus sativus*, eggplant *Solanum melongena*, cucumber *Cucumis sativus*, tomato *S. lycopersicum*, and chilli pepper *Capsicum annuum* [78,79]. Selenium fertilizers are already commercially available, at least from the Yara company (https://www.yara.co.uk/crop-nutrition/fertiliser/yara-booster-range/ accessed on 20 June 2023). The mineral composition contains SeNPs, Ca(OH)_2_NPs and oxidized steel nanoparticles and can be used to improve the condition of fruit plants during periods of drought [80]. Agricultural uses may also include protecting plants from insect larvae. There is evidence of larvicidal activity of SeNPs [81]. SeNPs can be also used in the development of heavy metal and hydrogen peroxide sensors for agriculture [82,83,84].

The seventh application is the development of drugs for the treatment of inflammatory diseases (for example, recovery from a stroke) or diabetes [85,86]. In particular, it was shown that Protein/SeNPs inhibited hydroxyl radical productions in vitro [87]. Cystein/SeNPs inhibited hyperglycemia-induced ROS production in HUVEC by 40–50% [88]. Data on preclinical trials of a drug for the treatment of DM2T based on liposomes containing SeNPs have been published [89] (Table 1).

Other popular non-metal nanoparticles are SiNPs. SiNPs have a number of interesting advantages [92,93].

The first advantage is cheap synthesis. SiNPs are synthesized by the microemulsion method in the presence of oils or by the Stobers method, which requires relatively available reagents [94,95]. SiNPs can also be synthesized by a mechanochemical method, while the raw material for the synthesis of SeNPs can be river sand [96].

The second advantage is a wide range of applications, including targeted drug delivery with very high specificity [97,98,99], bioimaging [100,101,102], the creation of sensors for the detection of glucose, narcotic substances, or nucleic acids [103,104,105], the up-conversion of light (improved photosynthesis processes in agricultural plants), the protection of cereals from drought [106], and the creation of materials with antibacterial properties. SiNPs have the ability to significantly modify the surface and composition to create SiNP-based nanocomposites with antimicrobial properties [107,108,109].

The third advantage is the practically absent toxicity against eukaryotic cells. Oral administration of 1000–2000 mg/kg SiNPs did not have toxic effects in in vivo experiments and did not cause damage to internal organs in rats [110,111].

The disadvantages of SiNPs include the complexity/high cost of surface modification or the addition of metal NPs and the difficulty of obtaining SiNPs with uniform characteristics [93]. As a rule, precious-metal NPs and/or modification by several agents at once are required [104,108,109]. Antimicrobial properties have not been described for SiNPs without surface modification or addition of metal NPs [92]. SeNPs have been described as having their own antimicrobial properties [112,113,114]. In addition, modified SeNPs with antimicrobial properties can be obtained by biosynthesis, which significantly reduces the cost of their production [115,116,117].

Recently, a series of reviews has been published on the antibacterial, antifungal, anticancer, antiviral and antiparasitic properties of SeNPs [118,119,120]. It should be noted that in these works, the main emphasis is on a detailed description of the antimicrobial mechanisms of SeNPs and the contribution of conjugates to the properties of SeNPs. However, the numerical dependences of the properties of SeNPs—n particular, their size and method of preparation—are described to a lesser extent. In addition, it remains unknown to what extent the “size-antimicrobial-property” dependences of SeNPs are preserved when moving from one type of microorganism to another.

In this literature review, we aimed to analyze the dependence of the antimicrobial effect of selenium nanoparticles on their size, features of synthesis and microorganism species such as viruses, bacteria and fungi. There is no doubt that the relevance of this topic is great.

## 2. Synthesis Methods of Selenium Nanoparticles

**Figure 3:**
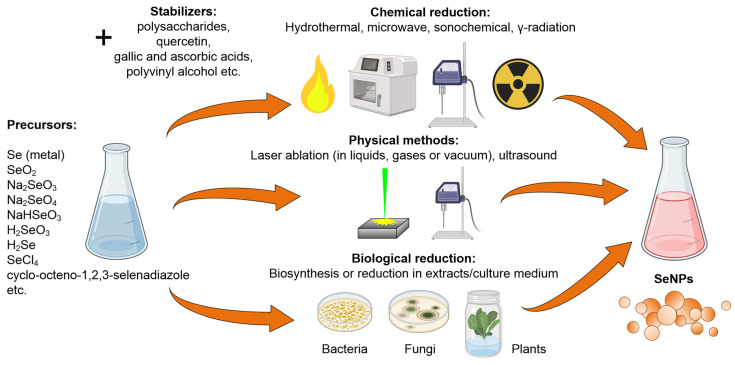
General approaches to SeNPs synthesis (references are given in the text).

In this subsection, we discuss various methods for the synthesis of selenium nanoparticles. SeNPs can be synthesized using a wide range of methods: sonochemical, reflux, microwave, hydrothermal, gamma irradiation, pulsed laser ablation, physical evaporation and “green synthesis” (biological reduction) (Figure 3). The most common method for selenium nanoparticle synthesis is chemical reduction [114]. The most commonly used precursors are SeO_2_, Na_2_SeO_3_, NaHSeO_3_ and H_2_SeO_3_ [121,122,123,124]. Less-common precursors are H_2_Se, Na_2_SeO_4_, SeCl_4_ or cyclo-octeno-1,2,3-selenadiazole [125,126,127,128].

As stabilizers, substances such as polysaccharides, quercetin, gallic and ascorbic acids and polyvinyl alcohol are used [129]. In addition to the stabilizer, a reducing agent (or reducer) is added to the solution, such as potassium tetrahydroborate (KBH_4_), ascorbic acid, hydrazine chloride, hydrazine hydrate (N_2_H_4_∙3H_2_O) or dimethylsulfoxide (C_2_H_6_OS) [130]. Sometimes substances of biological origin, mainly plant extracts, are used as reducing agents. This method of synthesis is called biological reduction [131,132]. The sonochemical method is a type of chemical reduction method in which the formation of metal NPs is formed by mixing soluble salts that react with each other to form a precipitate. Ultrasound accelerates of sediment formation. For exposure, a Ti tip immersed in a salt solution is used. It is noteworthy that, during precipitation in the presence of organic compounds, it is possible to obtain conjugated NPs [133]. The hydrothermal method is based on the reduction of inorganic precursors of SeNPs (Na_2_SO_3_) in aqueous solutions in the presence of organic reducing agents (for example, L-ascorbate) at an elevated temperature (~90 °C). An elevated temperature is necessary to accelerate the Se reduction reaction [134]. The reflux method is a variant of the chemical reduction method that occurs when the reaction mixture is boiled for a long time, which makes it very similar to the hydrothermal method. To increase the boiling time, a special unit with a refrigerant is used to condense the solvent vapors and return them to the reaction mixture [135]. The microwave method for the synthesis of nanoparticles is based on the reduction reaction of selenium-containing precursors in a medium with a reducing agent [136]. Microwave radiation provides a multiple acceleration of the reduction reaction due to heating of the reaction. The time of microwave exposure determines the type of crystal lattice of the synthesized Se nanoparticles [128,137]. In the method of gamma irradiation on the reduction of SeO_2_ to Se^0^ [138], recovery occurs due to processes associated with water radiolysis [139]. In addition, it has been shown that gamma radiation can enhance the biogenic synthesis of SeNPs using fungi [140,141]. Principally, the microwave method and the use of gamma radiation can be attributed to the group of chemical reduction methods, but we singled them out separately, since they additionally need high-energy electromagnetic sources.

Laser ablation in a liquid belongs to the class of physical synthesis methods. It usually consists of obtaining nanoparticles using intense laser radiation from the surface of massive crystalline selenium targets immersed in a liquid. The laser ablation method has been described in detail [142]. During further laser irradiation of a colloid with nanoparticles, it is possible to obtain smaller NPs. This process is called fragmentation [143]. Physical evaporation is a method for synthesizing nanoparticles by treating a metal target with a laser in a vacuum, inert gas or atmospheric air. The method is essentially laser ablation carried out not in a solution, but in a gas and/or vacuum [144,145], so these methods were combined in this work.

**Figure 4:**
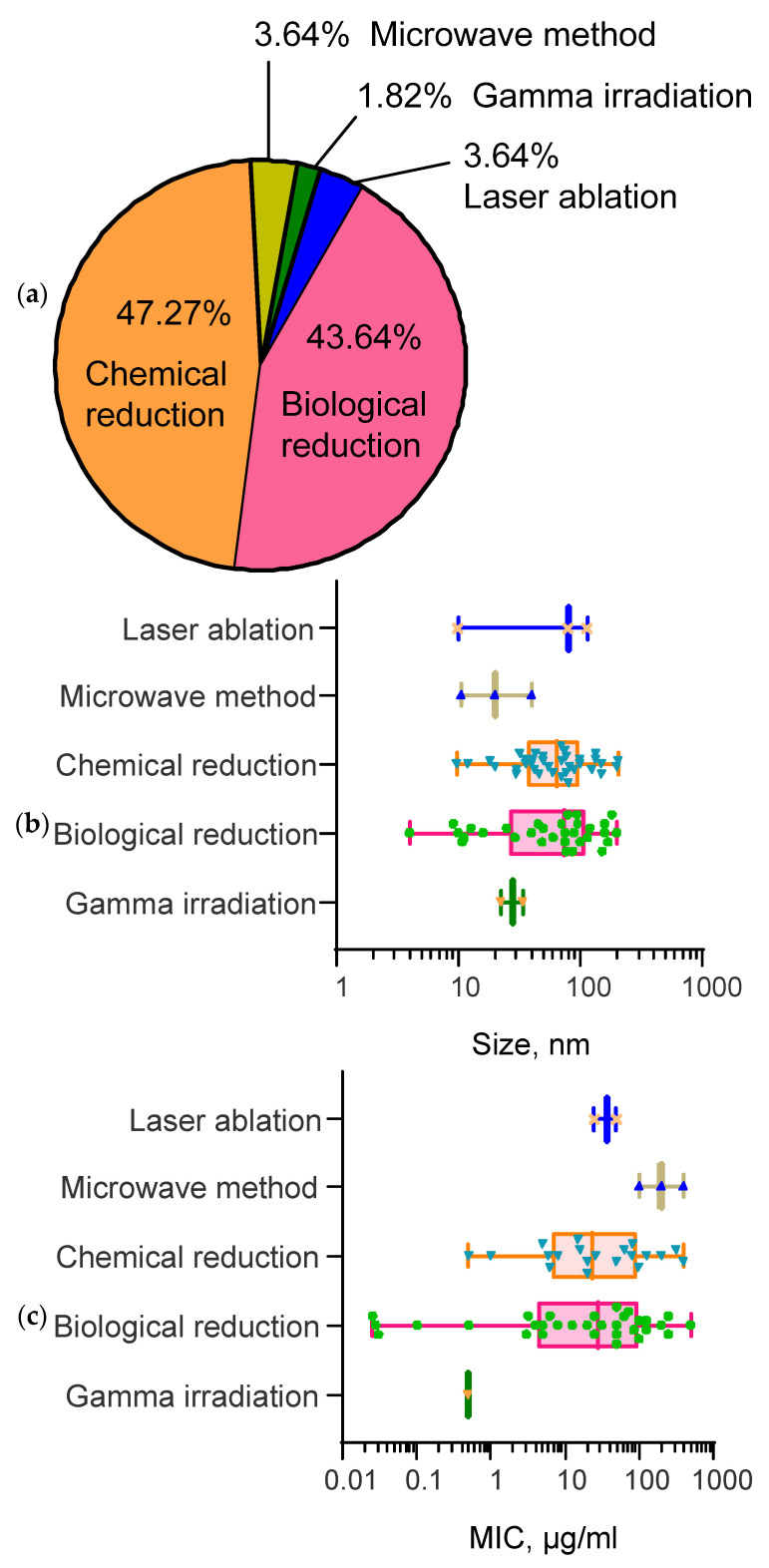
Influence of methods for selenium nanoparticle synthesis on the size and antibacterial properties of nanoparticles: (**a**) The ratio of the use of various methods for selenium nanoparticle synthesis according to the literature; (**b**) Size distribution of selenium nanoparticles for different types of synthesis; (**c**) Distribution of values of the minimum inhibitory concentration of selenium nanoparticles in different types of synthesis. Each symbol means the MIC value taken from a separate publication. Colors correspond to synthesis methods: orange crosses—laser ablation, blue triangles—microwave method, cyan triangles—chemical reduction, green squares—biological reduction, orange triangles—gamma irradiation.

In the case of the biological reduction method, biogenic synthesis and bioorganic synthesis are distinguished. In a significant number of works, the synthesis of SeNPs was carried out by biological reduction (“green synthesis”). This approach is also based on the reduction of selenium-containing precursors to Se^0^. The essential difference between the first and the second type is that biogenic synthesis uses cellular structures [115,131,146,147,148,149] and bioorganic synthesis uses noncellular extracts of plant and microbial origin to synthesize nanoparticles [117,132,150,151,152,153]. In this case, reducing agents are usually secondary metabolites, such as flavonoids, thiamine and capsaicin, as well as other agents containing amino groups, extracted from various plants: *Spirulina platensis*, *Azadirachta indica*, *Trigonella foenum-graecum*, *Allium sativum*, etc. [152,154,155,156,157]. The use of extracts during synthesis provides antibacterial, anticancer, or antioxidant properties of SeNPs [154,155,156,157]. SeNPs can be synthesized by cultivating microorganisms in a medium with an excess of SeNP precursors (sodium selenite (Na_2_SeO_3_) or SeO_2_) [158]. For the synthesis of SeNPs, biomass and/or cell-free supernatants of bacterial cultures (*Lactobacillus brevis*, *Lactobacillus casei*, *Bacillus licheniformis*, *Pseudomonas alcaliphila*, etc.) [158,159,160,161,162], fungi (*Aspergillus oryzae*, *Penicillium citrinum*, *Mariannaea* sp.) [140,141,163] or yeasts (*Saccharomyces cerevisiae*, *Magnusiomyces ingen*) [164,165] can be used. Figure 4a shows the percentages of the various methods for the synthesis of selenium nanoparticles found in the literature.

### 2.1. Influence of the Method of Synthesis of Selenium Nanoparticles on the Resulting Size and Shape of Nanoparticles

Does the method of synthesis of nanoparticles affect their size? The results of our analysis are presented in Figure 4b. Obviously, physical methods of synthesis, such as laser ablation or microwave irradiation, make it possible to achieve a narrow size distribution of nanoparticles (Figure 4b). Most often, these are spherical particles less than 200 nm. Chemical or biological synthesis methods produce a wide range of particle sizes from 5 to 500 nm (Figure 4b). Within the framework of one type of synthesis, a preparation of nanoparticles with a wide size distribution of nanoparticles is usually obtained. At the same time, the vast majority of publications have synthesized spherical selenium nanoparticles. In some cases, other shapes, such as elongated cylinders [166], polygons [167] and granules [146], are observed.

### 2.2. Influence of Selenium Nanoparticle Synthesis Method on the Minimum Inhibitory Concentration in Antibacterial Studies

It has been established that the selenium nanoparticle synthesis method affects the value of the minimum inhibitory concentration in antibacterial studies (Table 1). The results of the analysis are shown in Figure 4c. It was noted that when using physical methods for the synthesis of selenium nanoparticles, the minimum inhibited concentration for effective antibacterial action did not exceed 100 µg/mL. When using microwave generation of nanoparticles, the MIC is approximately 100–300 µg/mL, which is significantly worse compared with nanoparticles obtained by other methods.

It should be noted that there are few studies that use nanoparticles synthesized using physical methods. Most likely, in the future, we should expect clarification of the data presented. For the methods of chemical and biological synthesis, the distribution of the obtained values of the minimum inhibited concentration is quite wide. In some cases, MICs of less than 1 µg/mL have been reported. At the same time, the average efficiency of nanoparticles obtained by both chemical and biological synthesis does not differ significantly. In general, the average efficiency of nanoparticles obtained by chemical/biological methods and using laser ablation does not differ significantly.

One of the ways to enhance the antibacterial and antifungal properties of SeNPs is their functionalization with enzymes and polymers during synthesis or inclusion in a polymer matrix. Examples of such components can be: arabinogalactan, propolis, lysozyme, bacterial cellulose, gelatin, reduced graphene oxide, chitosan, collagen and ε-poly-L-lysine [121,125,168,169,170,171,172,173]. The addition of SeNPs to textile fiber significantly enhances their antimicrobial properties [122,174]. The use of plant extracts (*Urtica dioica*, *Ricinus communis*, *Artemisia annua*, *Nepeta* sp., etc.) during synthesis also enhances the antibacterial and antifungal effects of SeNPs [117,148,150,175]. The addition of SeNPs to titanium oxide nanotubes enhances the antibacterial properties of the latter [176,177,178].

## 3. Effective Concentration/Minimum Inhibitory Concentration of Selenium Nanoparticles Depending on Their Size

### 3.1. Dependence of the Effective Concentration of Selenium Nanoparticles on Their Size for the Study of Antiviral Activity

**Figure 5:**
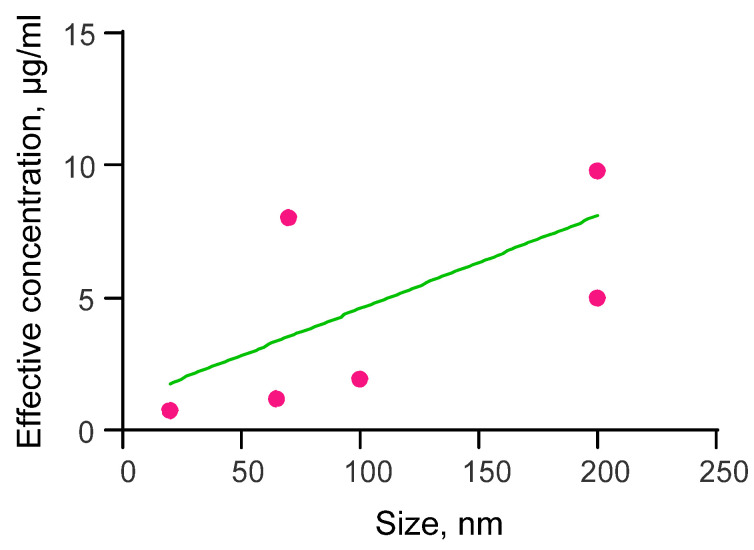
Dependence of the selenium nanoparticles’ effective concentration on their size in the study of antiviral activity (all results presented in the graph use selenium nanoparticles synthesized with the chemical reduction method). Each symbol means the MIC value taken from a separate publication. Colors correspond to type of susceptible microorganisms: red circles—antiviral activity. The green straight is the trend line. Data from Table 2 were used in Figure 4 and Figure 5.

We consider it necessary to start presenting the results with a study of antiviral activity. There are very few such research publications [120]. It seems that this is due to the increased complexity of organizing scientific work with viruses. Figure 5 shows the effective concentration of selenium nanoparticles depending on their size in the study of antiviral activity. The dependence is described with the equation y = 0.035·x + 1.08. By the nature of this dependence, it can be concluded that with a decrease in the size of nanoparticles, their effective concentration for antiviral action also decreases. This means that as the size of nanoparticles decreases, on average, they become more effective against viruses.

It should be noted that selenium nanoparticle synthesis, which is used in most antiviral studies, is a chemical reduction method. In the majority of research papers, spherical nanoparticles with a diameter of 10 to 200 nanometers were used. Published papers mainly use the H1N1 influenza virus (H1N1 influenza infecting Madin Darby canine kidney cell line) [179,180,181,182]. In addition, there are research publications investigating Enterovirus (Enterovirus 71—EV71) [183,184], hepatitis virus (HAV) [81,185,186], Cox-B4 virus (enteroviruses) [81], herpes virus (HSV-2 Herpes simplex II), influenza virus H1N1 [187] and adenovirus (Adenovirus strain 2) [187].

In the published research papers, two problems are investigated. The first task is to study the general antiviral action of selenium nanoparticles. The second task is to increase antiviral activity with the help of selenium nanoparticles in case of resistance of the virus to antiviral drugs. In other words, in the first task, researchers use “pure” selenium nanoparticles, and in the second task, selenium nanoparticles are processed (=functionalized) with the help of antiviral drug molecules. That is, selenium nanoparticles act as substrates for targeted delivery.

### 3.2. Dependence of the Minimum Inhibitory Concentration of Selenium Nanoparticles on Their Size and Shape in the Study of Antibacterial Activity

**Figure 6:**
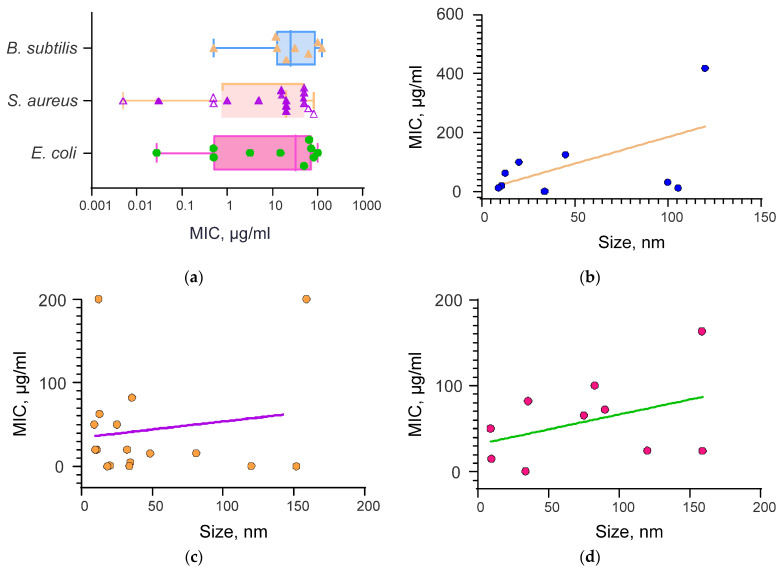
Antibacterial action of selenium nanoparticles. (**a**) Distribution of minimum inhibitory concentration (MIC) values of selenium nanoparticles for the three most common studied species of bacteria: *B. subtilis* (orange triangles), *S. aureus* (purple triangles), *E. coli* (green circles); (**b**–**d**) Dependence of the minimum inhibitory concentration of selenium nanoparticles on their size for the study of antibacterial activity: against *B. subtilis* (MIC values are blue circles, trend line is orange)—(**b**); *S. aureus* (MIC values are orange circles, trend line is purple)—(**c**); and *E. coli* bacteria (MIC values are red circles, trend line is green)—(**d**). Each symbol means the MIC value taken from a separate publication. Data from Table 2 were used in this figure.

A large number of studies are published on this topic every year. For convenience, we present Table 2, which contains the analyzed publications of the antibacterial action of selenium nanoparticles. It should be noted that in most published research papers, three types of bacteria are used as objects: *Bacillus subtilis*, *Staphylococcus aureus*, and *Escherichia coli*. A box-and-whisker plot of the values of the minimum inhibitory concentration for these three types of bacteria is shown in Figure 6a.

**Table 2:** Dependence of the antimicrobial properties of SeNPs on the size, composition and method of synthesis.

№	Precursor	Composition	Method of the Synthesis	Particle Size, nm	Microorganism Strains	Effect	MIC	Results	Reference
1	Na_2_SeO_3_	Lysozyme SeNPs	Chemical reduction	35.6	*Escherichia coli*, *Staphylococcus aureus*	BS	82 μg/mL	SeNPs and lysozyme demonstrated synergetic bacteriostatic activity.	[121]
2	Na_2_SeO_3_	Propolis SeNPs	Bioorganic chemical reduction	159, 151.9, 11.2 and 169.3	*Salmonella typhimurium* ATCC 14028, *Escherichia coli* ATCC 25922, *Staphylococcus aureus*- ATCC 25923	BC	25 mg/L 27.5 mg/L 30 mg/L	BNCt/Pro/SeNPs were the most effective against all bacterial strains.	[168]
3	Na_2_SeO_3_	SeNPs	Chemical reduction	32.3	*Staphylococcus aureus* (MSSA), *Staphylococcus aureus* (MRSA), *Staphylococcus aureus* (VRSA), *Enterococci* (VRE)	BC; BS	20 µg/mL, 80 µg/mL, 320 µg/mL, and >320 µg/mL	SeNPs showed a synergistic effect with linezolid (LZD) through protein degradation against MSSA and MRSA.	[112]
4	Na_2_SeO_3_	SeNPs	Biosynthesis of SeNPs by *Providencia* sp.	120	*P. aeruginosa*, *E. coli*, *V. parahemolyticus*, *S. aureus*, *B. cereus*, *B. subtilis*	BC; BS	500 mg/L	Bio-SeNPs showed strong antibacterial effects on the five of pathogens at 100 mg/L. It was shown that most of G-bacteria (*P. aeruginosa*, *E. coli* and *V. parahemolyticus*) were locally killed by 500 mg/L of the bio-SeNPs after 12 h, which was better than G+-bacteria (*S. aureus* and *B. cereus*, except for *B. subtilis*).	[188]
5	Se (solid)	SeNPs	Pulsed laser ablation in liquids	~80 and ~10	*E. coli* (MDR-EC) ATCC BAA-2471, *P. aeruginosa* (PA) ATCC 27853, *S. aureus* (MRSA) ATCC 4330 *Staphylococcus epidermidis* ATCC 35984	BC; BS	25 µg/mL	SeNPs showed a dose-dependent antibacterial effect toward both standard and antibiotic-resistant phenotypes of Gram-negative and Gram-positive bacteria.	[113]
6	NaHSeO_3_	SeNPs with polyester fabrics	Chemical reduction	40–60	*Salmonella typhi*, *Bacillus cereus*, *Escherichia coli*, *Pseudomonas aeruginosa*	BC	1980 µg/mL	The treated fabric under study showed excellent killing potentiality against Gram-positive and Gram-negative bacteria.	[174]
7	NaHSeO_3_	Leather material/ SeNPs	Chemical reduction	36–77 and 41–149	*Bacillus cereus*, *Pseudomonas aeruginosa*, *Salmonella typhi*, *Escherichia coli*	BC	1980 µg/mL	Potential application to the footwear industry to color the leather as well as prevent the spread of bacterial infection promoted by humidity, poor breathability and temperature.	[122]
8	Na_2_SeO_3_	SeNPs/ orange peel waste extract	Bioorganic chemical reduction	16–95	*Pseudomonas aeruginosa* PAO1, MDR, *S. aureus* ATCC 29213	BS	25 µg/mL	The biosynthesized SeNPs had a promising antibiofilm activity, where the largest inhibition of biofilm was noticed in MDR *K. pneumonia*.	[169]
9	Na_2_SeO_3_	bacterial cellulose/ gelatin/ SeNPs hydrogels	Chemical reduction	75	*E. coli*, *S. aureus*	BC	65.44 μg	BC/Gel/SeNPs nanocomposite hydrogel: potential wound dressing for preventing wound infection and accelerating skin regeneration in clinic.	[189]
10	H_2_SeO_3_	rGO-S/Se composite	Chemical reduction	12	*Staphylococcus aureus*, *Enterococcus faecalis*	BS	200 µg/mL	Concentration and time-dependent BS activity of the rGO (Reduced graphene oxide)-S/Se NP against *S. aureus* cells	[170]
11	Na_2_SeO_3_	SeNPs	Biosynthesis of SeNPs by cyanobacteria *Anabaena* sp.	25	*Staphylococcus aureus Escherichia coli*	BS	50 µg/mL	These biogenic SeNPs demonstrated significant antibacterial and anti-biofilm activity against bacterial pathogens.	[131]
12	Na_2_SeO_3_	Ag-SeNPs	Biosynthesis of SeNPs by *Aureobasidium pullulans*	50 and 70	*Staphylococcus aureus* F1557 *E. coli* WT F1693	BC; BS	-	The Ag–Se coating reduced 81.2% and 59.7% of viable bacterial adhesion. The antibacterial mechanism of Ag–Se coatings works through effective contact-killing activity against *S. aureus*.	[146]
13	Na_2_SeO_3_	SeNP-chitosan, SeNPs-carboxymethyl cellulose	Chemical reduction	55–500 50–300	*Staphylococcus aureus*, methicillin-resistant *Staphylococcus aureus*, *Staphylococcus epidermidis*	BS	5 µg/mL	The SeNP-modified collagenous scaffolds at SeNP concentrations low as 5 µg/mL showed a strong antibacterial effect (up to 94% of bacterial growth inhibition) toward laboratory and clinical isolates of Gram-positive bacteria from the genus Staphylococcus.	[171]
14	Na_2_SeO_3_	SeNPs	Biosynthesis from *Stenotrophomonas maltophilia* SeI TE02	181	*P. aeruginosa* PAO1, INT, BR1 and BR2, *S. maltophilia* VR10 and VR20, *Achromobacter xyloxidans strain C*, *Burkholderia cenocepacia* strain LMG 16656, *Staphylococcus aureus* Mu50 strain, *S. aureus* UR1, *Staphylococcus epidermidis* ET024, *Staphylococcus hemolitycus* UST1	BS	4–128 μg/mL	The progressive loss in protein and carbohydrate content of the organic cap determines a decrease in nanoparticle stability. This leads to an alteration of size and electrical properties of SeNPs along with a gradual attenuation of their antibacterial efficacy.	[190]
15	Na_2_SeO_4_	SeNPs	Biosynthesis of SeNPs from *Aspergillus quadrilineatus* and *Aspergillus ochraceus* isolated from the twigs and leaves of *Ricinus communis*	45–75	*Pseudomonas aeruginosa* ATCC 15442, *Bacillus cereus* ATCC 10876, *Staphylococcus aureus* ATCC 6538, *Klebsiella pneumoniae* ATCC 13883, *Bacillus subtilis* TCC 6633, *Escherichia coli* ATCC 11229	BS	62.5–1000 µg/mL	SeNPs showed potent antifungal and antibacterial potentials against different human and phyto-pathogens.	[175]
16	Na_2_SeO_3_	“Green” SeNPs	Biosynthesis using aqueous leaf extract of *U. dioica*	10–87.4	*Staphylococcus aureus* ATCC 25923, *Bacillus subtilis* ATCC605, *Escherichia coli* ATCC 25922, *Pseudomonas aeruginosa* ATCC 27853 Fungi: *Candida albicans*, *Cryptococcus neoformans*	BS, FS	125, 62.5 and 15.62 µg/mL 3.9 and 7.81 µg/mL	SeNPs exhibited promising antibacterial activity against Gram-negative and Gram-positive bacteria and antifungal activity.	[150]
17	SeO_2_	SeNPs/tree gum	Chemical reduction	105.6	*Bacillus subtilis*, *Micrococcus luteus*	BS	12 μg/mL	The synthesized SeNPs inhibited the growth of the Gram-positive bacteria *B. subtilis* only.	[123]
18	Na_2_SeO_3_	SeNPs/chitosan	Chemical reduction	100	*Staphylococcus aureus*, *Escherichia coli*	BS	158 μg/mL	The antibacterial activity of CS(H)-SeNPs markedly decreased owing to the aggregation of NPs.	[166]
19	Na_2_SeO_3_	SeNPs	Phytofabrication of SeNPs from aqueous *Spirulina platensis*	79.4	*Salmonella abony* NCTC 6017, *Klebsiella pneumonia* ATCC 700603, *E. coli* ATCC 8739	BS	25–200 µg/mL	SeNPs have shown potent antimicrobial activity against Gram-negative bacteria. No toxic effect was observed for SeNPs on normal kidney and liver cell lines.	[191]
20	Na_2_SeO_3_	SeNPs	Biosynthesis of SeNPs from *Nepeta* plant powder	75	*P. aeruginosa*: ATCC 27853 and *A. baumannii*: ATCC BAA-747	BS	4 μg/mL 8 μg/mL	The inhibition of bacterial growth demonstrated in the presence of lower concentrations of SeNPs than common antibiotics.	[117]
21	Na_2_SeO_3_	Collagen/Chitosan/SeNPs	Chemical reduction	100–200	*Staphylococcus aureus* NCTC 8511, MRSA CCM 7110 and *Escherichia coli* NCTC 13216	BS	0.5–5 µg/mL	SeNPs are able to enhance the scaffold’s antibacterial properties toward *S. aureus* and MRSA at concentrations between 0.5 µg/mL and 5 µg/mL.	[172]
22	Na_2_SeO_3_	B-SeNPs	Biosynthesis of SeNPs from *Anabaena variabilis* (cyanobacteria)	10.8	*Bacillus subtilis*, *Staphylococcus aureus*, *Escherichia coli*, *Klebsiella pneumoniae*	BS	20 µg/mL	Cyanobacteria mediated synthesis can be considered as safe and nontoxic way to synthesize SeNPs.	[116]
23	Na_2_SeO_3_	SeNPs	Biosynthesis of SeNPs-S by *Bacillus* sp. Q33	159.2	*E. coli*, *P. aeruginosa*, *S. aureus*, *L. monocytogenes*	BS	200 µg/mL	SeNPs-S (product of whole cells) and SeNPs-E (product of the extracellular extract) exhibited obvious inhibitory effects on the four pathogenic bacteria.	[115]
24	Se (wafer)	SeNPs	Chemical reduction	42	*E. coli*, *S. aureus*	BS	0.2 mg/mL	The synergistic antibacterial effect of SeNPs and microstructured parylene-C.	[192]
25	H_2_Se	Arabinogalactan/ SeNPs	Bioorganic synthesis with AG from *Larix Sibirica* isolated	94	bacterial phytopathogen *Clavibacter michiganensis sepedonicus* (Cms)	BS	6.25 μg/mL	Antimicrobial activity of AG/SeNPs is due to their ability to inhibit the dehydrogenase activity of Cms cells, to disrupt the integrity of the cell membrane, resulting in a decrease of transmembrane potential and reduction of cellular respiration.	[125]
26	Na_2_SeO_3_	Cefotaxime/Ag–SeNPs	Gamma irradiation	34.5; 24.9	*E. coli*, *P. aeruginosa*, *K. pneumoniae*, *S. aureus*, *Enterococcus* sp.	BS; BC	2.5–5 μg/mL; 0.625–2.5 μg/mL (with CFM)	Ag NPs-CFM, SeNPs-CFM and Ag–SeNPs-CFM possessed antimicrobial activity against *Staphylococcus aureus*, *Escherichia coli*.	[193]
27	Na_2_SeO_3_s	SeNPs	Chemical reduction	70	*Porphyromonas gingivalis*	BS; BC	4–16 μg/mL	The growth of *P. gingivalis* was significantly inhibited by SeNPs.	[114]
28	SeO_2_	Se NP-ε-poly-L-lysine	chemical reduction	82	*S. aureus* ATCC 29213, *S. aureus* (MRSA) ATCC 43300, *E. faecalis* ATCC 29212, *E. coli* ATCC 25922, *A. baumannii* 2208, ATCC 19606, *P. aeruginosa* strain PAO1-LAC ATCC 47085, *K. pneumoniae* ATCC 13883, and *K. pneumoniae* (MDR) FADDI-KP628	BS; BC	6−26 μg/mL	The MICs of Se NP-ε-PL against the eight different types of bacteria tested are approximately 6–26 μg/mL.	[173]
29	Na_2_SeO_3_	SeNPs	Biosynthesis by Se-resistant *Bacillus subtilis* AS12	77	*Aeromonas hydrophilia* *Staphylococcus aureus*, *Bacillus cereus*, *Listeria monocytogenes*, *Escherichia coli*, *Aeromonas hydrophilia*, *Klebsiella pneumonia*	BS; BC	3–5 μg/mL	Bio-SeNPs can mitigate the accumulation of heavy metals and reduce the bacterial load in a concentration-dependent manner.	[194]
30	Na_2_SeO_3_	TiO_2_ nanotube with SeNPs	chemical reduction	88.93	*E. coli*	BS	-	Selenium nanoparticles improved antibacterial properties of titanium dioxide nanotubes.	[176]
31	Na_2_SeO_3_	SeNPs	microwave technique in the presence of citric acid	10.5–20	*P. aeruginosa*, *E. coli*, *B. subtilis*, *S. aureus*	BS	100 mg/mL	SeNPs had the highest activity against *E. coli*, with a zone of inhibition (ZOI) of 25.2 ± 1.5 mm compared to 16.0 ± 0.6 mm for the standard antibiotic.	[136]
32	Na_2_SeO_3_	SeNPs	biosynthesis of SeNPs by endophytic fungal strain *Penicillium crustosum* EP-1	3–22	*Bacillus subtilis* ATCC 6633, *Staphylococcus aureus* ATCC 6538, *Escherichia coli* ATCC 8739, *Pseudomonas aeruginosa* ATCC 9022	BS	12.5 µg/mL 50 µg/mL 50 µg/mL 25 µg/mL (in the presence of light) 50 µg/mL, 100 µg/mL (under dark conditions)	The effect of SeNPs was dose-dependent, and higher activities against bacteria were attained in the presence of light than were attained under dark conditions.	[147]
33	Na_2_SeO_3_	Mk-SeNPs	chemical reduction with the presence of aqueous berry extract of *Murraya koenigii* (Mk-SeNPs)	50–150	*Streptococcus mutans* (HQ 693279.1 & ATCC 25175), *Enterococcus faecalis Shigella sonnei*, *Pseudomonas aeruginosa* (K 7769531 & HQ 693272	BS; BC	40 μg/mL 50 μg/mL	Mk-SeNPs are considered to be a prospective antibacterial agent with effective antioxidant capacity at 25 and 50 μg/mL, which is target-specific only for the bacterial cells and not for the erythrocytes and macrophages at the same concentration.	[195]
34	Na_2_SeO_3_	SeNPs	The abiotic reduction of selenite with the use of *Enterococcus* spp. cell-free extract (biotic and abiotic stages)	200	*E. coli*	BS	3.2 g/L	The obtained nanoparticles exhibited antimicrobial properties by directly inhibiting the viability of an *E. coli* bacterial strain. The results demonstrate not only the potential of abiotic production of SeNPs but also the potential for these particles as microbial inhibitors in medical or similar fields.	[196]
35	Na_2_SeO_3_	SeNPs	chemical reduction with PVA as a stabilizer	30–70	*S. aureus* (ATCC 29213), *E. coli* (ATCC 25922)	BS	1 μg/mL	The growth of *S. aureus* was inhibited by the nanoparticles at concentrations as low as 1 μg/mL.	[197]
36	Na_2_SeO_3_	*Artemisia annua* extract/SeNPs	Biosynthesized using *Artemisia* *annua*, and subsequently, the surface of the biogenic SeNPs was functionally modified with starch.	<200	*Staphylococcus aureus*, *Bacillus cereus*, *Salmonella enterica*, *Escherichia coli*	BS; BC	5–100 μg/mL	StAaSeNPs showed the highest antibacterial activity against tested strains *S. enterica* (23.26 ± 0.35 mm). Based on the findings, it can be inferred that surface chemistry is the most influential factor in determining the antibacterial activity of SeNPs.	[148]
37	Na_2_SeO_3_	Hollow SeNPs	Bioorganic synthesis of SeNPs with the potato extract	115	*B. subtilis* (MTCC441), *E. coli* (MTCC40)	BC	10–20 μg/mL	The hSeNPs showed good antibacterial activity against tested bacteria.	[198]
38	SeO_2_	SeNPs	chemical reduction with the presence of PVA	43–205	*Staphylococcus aureus* (MSSA) ATCC 29213, *Staphylococcus aureus* (MRSA) ATCC 43300	BC	16 µg/mL	The SeNPs were shown to have multimodal mechanisms of action that depended on their size, including depleting internal ATP, inducing ROS production, and disrupting membrane potential.	[199]
39	Na_2_SeO_3_	BSA/ SeNPs	chemical reduction method in the presence of the BSA	20–30	*Escherichia coli* (ATCC no. 25922), *Escherichia coli* (ATCC no. BAA-2471), *Staphylococcus aureus* (ATCC no. 25923)	BC	1 mg/mL	SeNPs achieved a 10-fold reduction for *S. aureus.*	[200]
40	SeO_2_	eADF4(κ16)/SeNPs PVA/ SeNPs	chemical reduction method in the presence of the spider silk protein eADF4(κ16) and PVA (Polyvinyl alcohol)	46	*Escherichia coli*	BC	8 ± 1 µg/mL 405 ± 80 µg/mL	The eADF4(κ16)-coated SeNPs demonstrated a much higher bactericidal efficacy against the *E. coli*, with a minimum bactericidal concentration (MBC) approximately 50 times lower than that of PVA/SeNPs.	[201]
41	Na_2_SeO_3_	mycogenic SeNPs SeNPs-CN	2 methods: a biogenic process using *Penicillium chrysogenum* filtrate and by utilizing gentamicin drug (CN) following the application of gamma irradiation	33.84 22.37	*Staphylococcus aureus*, *Bacillus subtilis*, *Pseudomonas aeruginosa*, *Escherichia coli*, *Klebsiella pneumoniae* Fungi: *Candida albicans*	BC, FC	0.490 μg/mL 0.245 μg/mL	The synthesized SeNPs-CN possesses an encouraging antimicrobial potential with respect to the biogenic SeNPs against all examined microbes. Remarkably, SeNPs-CN showed antimicrobial potential toward 23.0 mm ZOI for *Escherichia coli* and 20.0 mm ZOI against *Staphylococcus aureus*. It also inhibited the expansion and invasion of *C. albicans* suggested the use of gentamycin as antifungal agent after the combination with the synthesized SeNPs.	[202]
42	Se (pellets)	SeNPs	Pulsed laser ablation in liquids	115	*Escherichia coli*, *Staphylococcus aureus*	BC	50 µg/mL	The pure selenium nanoparticles determined the minimal concentration required for ~50% inhibition of either *E. coli* or *S. aureus* after 24 h to be at least ~50 µg/mL.	[203]
43	H_2_SeO_3_	Green Orange Peel extract/SeNPs	Chemical reduction in the presence of BSA—stabilizer	18.3	*S. aureus* (ATCC 25923), *S. epidermidis* (ATCC 1228)	BS	4.94 μg/L	The SeNP sample demonstrated excellent antibacterial activity with an average diameter of inhibition zones of 20.0 mm and an MIC of 4.94 μg/L.	[124]
44	Na_2_SeO_3_	SeNPs	Bioorganic synthesis of SeNPs with the use of *Penicillium corylophilum* As-1 biomass filtrate, in presence of ascorbic acid as a reducing agent	29.1–48.9	*Staphylococcus aureus* ATCC 6538, *Bacillus subtilis* ATCC 6633, *Escherichia coli* ATCC 8739, *Pseudomonas aeruginosa* ATCC 9027	BS	9.37 μg/mL 18.75 μg/mL 37.5 μg/mL 37.5 μg/mL	The formed SeNPs showed a prominent antimicrobial activity at different concentrations against the pathogens *Staphylococcus aureus*, *Bacillus subtilis*, *Pseudomonas aeruginosa* and *E. coli*.	[132]
45	SeO_2_	*Penicillium expansum*/SeNPs	biosynthesis with *Penicillium expansum* ATTC 36200	4–12.7	*Bacillus subtilis* ATCC 6051, *Staphylococcus aureus* ATCC 23235, *Escherichia coli* ATCC 8739, *Pseudomonas aeruginosa* ATCC 9027 Fungi: *Candida albicans* ATCC 90028, *Aspergillus fumigatus* RCMB 02568, *A. niger* RCMB 02724	BS, FS	62.5 μg/mL 62.5 μg/mL 125 μg/mL 125 μg/mL 125 μg/mL 125 μg/mL 125 μg/mL	The inhibitory effect against Gram-positive bacteria was more pronounced than against Gram-negative bacteria and fungi.	[149]
46	Na_2_SeO_3_	Chitosan/SeNPs	chemical reduction	77	*Streptococcus mutans*	BS	128 and 64 µg/mL	The comparison between the treated and untreated groups showed that combining therapy with SeNPs and PDT markedly decreased colony-forming units of one-day-old *S. mutans* biofilm.	[204]
47	SeO_2_	SeNPs	solvothermal method using *Moringa oleifera* leaf extract as a reducing agent	82.86	*Listeria innocua* ATCC 33090, *Bacillus cereus* ATCC 10876, *Escherichia coli* ATCC 43888, *Salmonella typhimurium* ATCC 14028	BS	100 μg/mL	Zones of inhibition were observed only in *S. typhimurium* (12.5 ± 0.5 mm), *E. coli* (10.1 ± 0.7 mm) and *B. cereus* (9.8 ± 0.7 mm).	[167]
48	Na_2_SeO_3_	SeNPs	green synthesis using ascorbic acid as a reducing agent and methanolic extract of *Calendula officinalis L*. flowers as a stabilizer	40–60	*Serratia marcescens*, *Enterobacter cloacae*, *Alcaligenes faecalis*	BS	-	The antibacterial activity of the extract, AsAc, and Na_2_SeO_3_ was enhanced by producing the SeNPs, which significantly inhibited the growth of *S. marcescens*, *E. cloacae*, and *A. faecalis* bacterial strains.	[205]
49	Na_2_SeO_3_	SeNPs	Chemical reduction synthesis from extracts of three plants: *Allium cepa* (onion), *Malpighia emarginata* (acerola), and *Gymnanthemum amygdalinum* (boldo)	245–321	*Streptococcus agalactiae*, *Staphylococcus aureus*, *S. aureus*, *Pseudomonas aeruginosa*, *Escherichia coli*	BS	6.125 to 98 μg/mL	The antimicrobial activity and low hemolytic concentration indicate the possibility of use against Gram-positive bacteria, including multidrug-resistant ones, opening a wide variety of options for their application	[151]
50	SeO_2_	Algae/ SeNPs	Microwave-assisted synthesis of SeNPs	40	*V. harveyi* (PTCC 1755)	BS	200 μg/mL	The presence of different functional groups of *Sargassum angustifolium* on the surface of the algae-coated SeNPs might be responsible for the more effective reaction of these nanoparticles with the cell walls and/or membrane of *V. harveyi.*	[206]
51	Na_2_SeO_3_	SeNPs	chemical reduction	71	*V. cholerae* O1 ATCC 14035 strain	BS	50–200 μg/mL	SeNPs are safe as an antibacterial and antibiofilm agent against *V. cholerae* O1 ATCC 14035 strain.	[207]
52	Na_2_SeO_3_	SeNPs, NCT/GA, NCT/GA/Eug and NCT/GA/Eug/SeNPs	chemical reduction	9.7, 124.8, 132.6 and 134.2 nm	*Escherichia coli*, *Staphylococcus aureus*	BS; BC	15.0 μg/mL 20 μg/mL	The entire fabricated nanocomposite exhibited potent antibacterial activity and cell destruction capability within 5–10 h of exposure.	[208]
53	Na_2_SeO_3_	SeNPs	synthesis and purification, in the presence of pepper extract; chemical reduction method, plus microwave	79–90 nm	*Escherichia coli* ATCC BAA-2471, *Staphylococcus aureus* ATCC 4330	BS	72,2 μg/mL 85,1 μg/mL	Selenium nanoparticles were biocompatible and showed bacteriostatic activity	[152]
54	Na_2_SeO_3_	SeNPs	biosynthesized with a standard strain of *C. albicans*	38	Fungi: *Candida albicans*, *Candida glabrata*	FS	1 and 0.5 µg/mL	SeNPs showed much better fungistatic activity compared to itraconazole, amphotericin B and anidulafungin.	[209]
55	Na_2_SeO_3_	SeNPs	Biosynthesis from lactic acid bacteria (LAB)	56	Fungi: *Candida* and *Fusarium species*	FC	80–130 µg/mL	The LAB-SeNPs MFC was in the range of 80–130 µg/mL, which ensured the complete killing of all tested fungi.	[210]
56	Na_2_SeO_4_	SeNPs	Biosynthesis with standard strains of *A. Flavus* and *C. albicans*	37 and 38	Fungi: *Candida* and *Aspergillus species*	FS	0.5, and 0.25 μg/mL	The utilization of SeNPs at concentrations of 1, 0.5 and 0.25 μg/mL or in, some strains, even lesss than 0.125 μg/mL, resulted in zero growth of fungal agents.	[126]
57	Na_2_SeO_3_	SeNPs	“green” method using the *Halomonas elongata* bacterium	11	Fungi: *Candida albicans*	FS	-	The synthesized NPs in optimal situation stopped the growth of *Candida albicans* up to 72%.	[211]
58	Se (pellets)	Chitosan/SeNPs	laser ablation in liquids	100	Fungi: *C. albicans* TW17 and 6486 strains	FC, FS	3.5 μg/mL	Taken separately, SeNPs and CS have shown fungicidal properties, but when combined (CS-SeNPs), achieved a potent inhibitory effect against the mature biofilm in a dose–response manner.	[212]
59	Na_2_SeO_3_	SeNPs	biosynthesis with the leaf extract of *Melia azedarach*	74	Fungi: *Fusarium mangiferae*	FC	300 μg/mL	Biogenic selenium NPs are widely expected to be efficient and cost-effective treatments for fungal plant diseases.	[213]
60	Na_2_SeO_3_	SeNPs	green synthesis using extracts from *A. glaucum* leaves and *C. officinalis* flowers	8 and 133	Fungi: *Fusarium oxysporum*, *Colletotrichum gloeosporioides*	FS	0.25 mg/mL	It was observed that both SeNPs had antifungal activity against both plant pathogens at concentrations of 0.25 mg/mL and above. SeNPs-AGL demonstrated better antifungal activity and smaller size (approximately 8.0 nm) than SeNPs-COF (134.0 nm).	[214]
61	Na_2_SeO_3_	SeNPs	biosynthesis by *Lactobacillus aci-* *dophilus* ML1	46	Fungi: *Fusarium culmorum*, *Fusarium graminearum*	FC	100 mg/mL	Under greenhouse conditions, the wheat supplemented with BioSeNPs (100 mg/mL) experienced significantly incidence of crown and root rot diseases by 75% and considerably enhanced plant growth, grain quantity and quality by 5–40%.	[215]
62	SeCl_4_	SeNPs	biosynthesis using endophytic fungus *Fusarium oxysporum*	42	Fungi: *Aspergillus niger*	FS	8 mg/mL; diluted to 4, 2, 1, 0.5, and 0.25 mg/mL	SeNPs showed excellent antifungal and antisporulant activity against black fungus *Aspergillus niger*, which has become life-threatening to SARS-CoV-2 patients during the pandemic.	[127]
63	Na_2_SeO_4_	SeNPs	biosynthesis with the use of *Aspergillus* strains	64.8	Fungi: *Aspergillus fumigatus*, *Aspergillus flavus*	FS	0.5 µg/mL	The MIC of itraconazole and amphotericin B against A. fumigatus and A. flavus was 4 μg/mL, whereas the MIC values for treated samples with SeNPs have decreased to 0.5 μg/mL.	[216]
64	Na_2_SeO_3_	SeNPs	biosynthesis using the extract of Melia azedarach leaves	61	Fungi: *Puccinia striformis*	FS	30 mg/L	SeNPs at a concentration of 30 mg/L reduced the disease severity and enhanced the morphological, physiological, biochemical and antioxidant parameters.	[217]
65	Na_2_SeO_3_	PPE/SeNPs and NCT/PPE/SeNPs	Pomegranate peel extract (PPE) used for biosynthesis	9.4 85	Fungi: *Penicillium digitatum*	FC	22.5 15 mg/mL	NCT/PPE/SeNPs nanocomposite was the most effective and significantly exceeded the fungicidal action of standard fungicide. The direct treatment of fungal mycelia with NCT/PPE/SeNPs nanocomposite led to remarkable lysis and deformations of *P. digitatum* hyphae within 12 h of treatment.	[218]
66	H_2_SeO_3_	SeNPs	Biosynthesis by *Bacillus megaterium* ATCC 55000	41.2	Fungi: *Rhizoctonia solani* RCMB 031001	FS, FC	0.0625 and 1 mM	SeNPs improve morphological and metabolic indicators and yield significantly compared with infected control.	[219]
67	Na_2_SeO_3_	SeNPs	chemical reduction method with the use of the *Trichoderma* *atroviride* cell culture lysate	93.2–98.5	Fungi: *Pyricularia grisea*, *Colletotrichum capsici*, *Alternaria solani* on chili and tomato leaves	FS	50 μg/mL 100 μg/mL 100 μg/mL	The synthesized nanoparticles displayed excellent in vitro antifungal activity against *Pyricularia grisea* and inhibited the infection of *Colletotrichum capsici* and *Alternaria solani* on chili and tomato leaves.	[220]
68	Na_2_SeO_3_	bovine serum albumin (BSA)/ SeNP, ascorbic acid/)/ SeNP, chitosan/SeNP, glucose/SeNP	chemical reduction method	70–300	*Staphylococcus aureus* (ATCC 6538), *Enterococcus faecalis* (ATCC 29212), *Bacillus subtilis* (ATCC 6633), and *Kocuria rhizophila* (ATCC 9341), *Escherichia coli* (ATCC 8739), *Salmonella* sp. (NCTC 6017), *Klebsiella pneumoniae* (NCIMB 9111), *Pseudomonas aeruginosa* (ATCC 9027), Fungi: *Candida albicans* (ATCC 10231)	BS, BC, FC	100 μg/mL 100 μg/mL 200 μg/mL 200 μg/mL 400 μg/mL 400 μg/mL 200 μg/mL 400 μg/mL 25 μg/mL	Chitosan/SeNPs had greater antibacterial and antifungal activity than BSA/SeNPs and glucose/SeNPs. The MIC for Gram-positive bacteria was higher.	[221]
69	Na_2_SeO_3_	TiO_2_-nanotubes/ SeNPs, AgNPs or Ag_2_SeNP	electrolysis of Na_2_SO_3_	<10	*Staphylococcus epidermidis*	BS	-	Nanocomposite reduced bacterial growth and biofilm formation. In comparison with the non-modifed control, the TiO_2_-nanotubes/SeNPs surfaces showed a signifcantly higher coverage area with osteoblastic MG-63-cells.	[177]
70	Na_2_SeO_3_	TiO_2_-nanotubes/ SeNPs	Chemical reduction in presence of TiO_2_-nanotubes	<10	*Escherichia coli*, *Staphylococcus aureus*	BS	-	Samples reduced the density of *E. coli* by 94.6% and of *S. aureus* by 89.6% compared to titanium controls.	[178]
71	Na_2_SeO_3_	polycarbonate films/SeNPs	Chemical reduction by glutation	50–100	*Staphylococcus aureus*	BS		Polycarbonate films/SeNPs inhibited bacterial growth to 8.9% and 27% when compared with an uncoated polycarbonate surface after 24 and 72 h, respectively.	

BC—bactericidal effect, BS—bacteriostatic effect, FC—fungicidal effect, FS—fungistatic effect.

Se nanospheres are described in a significant portion of the studies [117,121,122,123,168]. However, other forms of SeNPs are described in a number of works: nanowires, nanorods and nanotubes. The form of SeNPs depends on the method and conditions of synthesis (pH, the presence and nature of the conjugate) [112,222]. Nanowires have bacteriostatic activity, but their MIC is comparable to or ~4–16 times lower than that of spherical SeNPs [112,222]. Comparable or higher MICs against bacteria and fungi were also found for Se nanorods compared with spherical SeNPs [112,222]. The literature also describes selenium-containing nanotubular structures with antibacterial activity [221,222]. However, it is difficult to compare the data of these works with the rest, since it is not possible to accurately estimate the final concentration of SeNPs in the obtained composites. The works devoted to the synthesis of “true” Se nanotubes are few and describe their potential application in technology, for example, in the creation of photosensors or solar cells; there are practically no works on antimicrobial applications of Se nanotubes [223].

Using regression analysis, it was found that the smaller the size of the selenium nanoparticles, the lower the necessary concentration of selenium nanoparticles for effective inhibition of bacterial growth (Figure 6b–d). Graphs are described by the following equations: y = 0.35x + 31.75 for the bacterium *E. coli*; y = 1.8x + 4.55 for the bacterium *B. subtilis*; and y = 0.19x + 34.65 for the bacterium *S. aureus*. For the bacterium *B. subtilis*, the regression coefficient was 1.8 (Figure 6c); for the bacterium *E. coli*, it was 0.35 (Figure 6b); and for the bacterium *S. aureus*, the lowest value was 0.19 (Figure 6d). In other words, a more efficient relationship between size and MIC was observed for *B. subtilis* than for *E. coli* and *S. aureus*.

A number of studies have shown that SeNPs have different antibacterial activities against Gram-positive and Gram-negative bacteria. A more pronounced antibacterial effect of SeNPs against Gram-negative bacteria compared to Gram-positive bacteria was shown. This property is of particular interest because, at present, among the bacteria that cause bacteremia, including sepsis, the proportion of Gram-negative bacteria is significantly increasing [224].

### 3.3. Dependence of the Minimum Inhibitory Concentration of Selenium Nanoparticles on Their Size in the Study of Antifungal Activity

Data were analyzed not only on the antiviral and antibacterial but also on the antifungal effect of selenium nanoparticles. Many more research papers have been devoted to the antifungal effect of selenium nanoparticles than to the antiviral effect; however, this effect is less than the antibacterial effect. In most fungal studies, the following species are used: *Candida* [126,150,202,209,210,211,212] and *Fusarium* [210,213,214,215]. Apart from these, the following fungal species are used: *Colletotrichum* [214,220], *Puccinia* [217], *Aspergillus* [127,216], *Cryptococcus* [150], *Penicillium* [218], *Rhizoctonia* [219], *Pyricularia* [220] and *Alternaria* [220]. Data on articles taken for analysis are also presented in Table 2.

**Figure 7:**
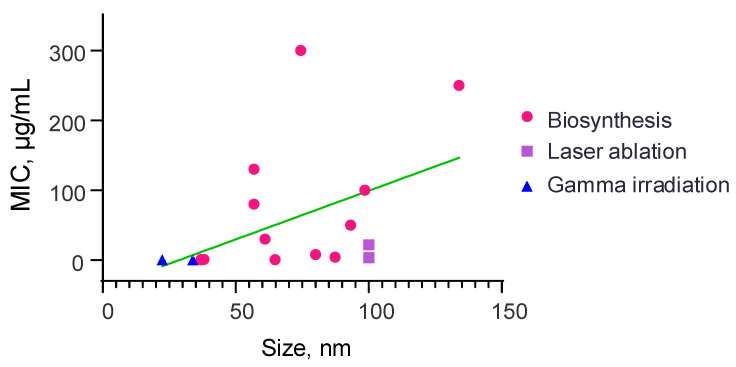
Dependence of the minimum inhibitory concentration of selenium nanoparticles on their size in the study of antifungal activity. Preparations of nanoparticles synthesized using gamma irradiation are marked in blue, purple—using the method of laser ablation in a liquid, pink—using biosynthesis.

Figure 7 shows the dependence of the minimum inhibitory concentration on the size of the nanoparticles. Using regression analysis, we can conclude that the smaller the size of the nanoparticles, the lower the value of the minimum inhibitory concentration (dependence equation y = 1.39x − 40.11). The graph shows the methods by which selenium nanoparticle synthesis was obtained. Most of the analyzed results use nanoparticles obtained by biological synthesis. We have presented research papers that use physical synthesis methods (laser ablation, gamma irradiation) to obtain selenium nanoparticles to study their antifungal effect. The nanoparticles used in these articles are usually spherical, and their size varies from 20 to 130 nm.

## 4. Mechanisms of Selenium Nanoparticle Antimicrobial Action

**Figure 8:**
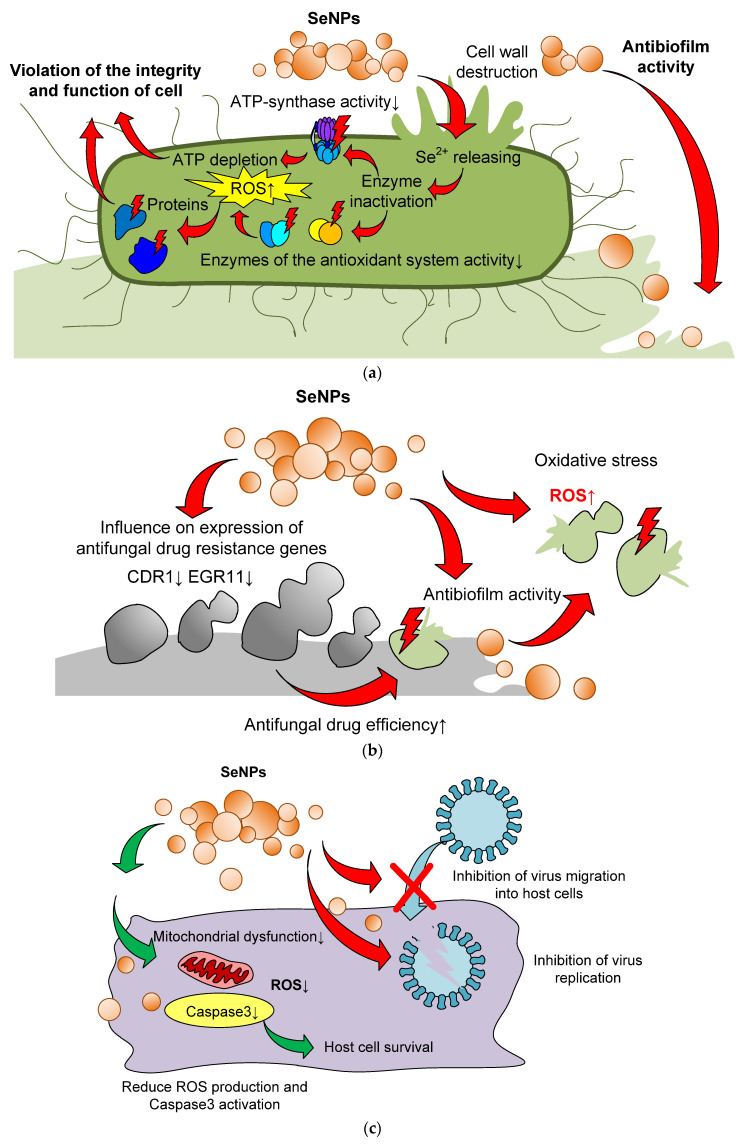

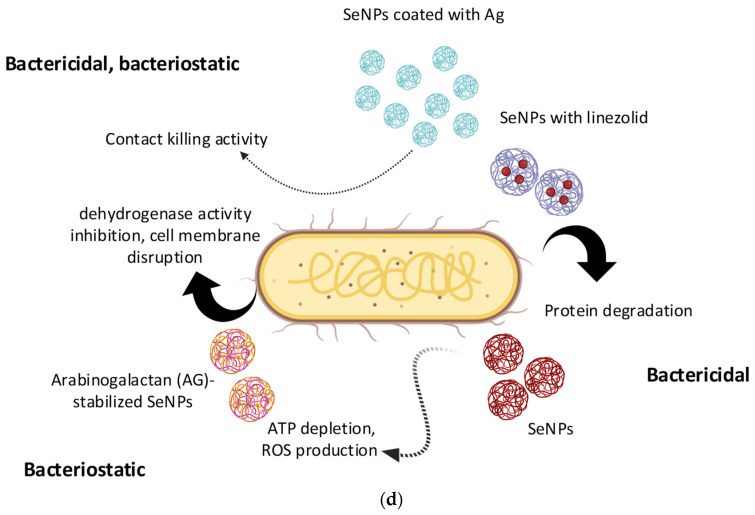
Mechanisms of SeNPs’ antibacterial (**a**), antifungal (**b**) and antiviral (**c**) actions. Effect of SeNP functionalization on antibacterial activity of SeNPs (**d**). The arrows show the causal relationship of events. Red and black arrows indicate effects on microorganisms. Green arrows show effects on eukaryotic cells.

We decided to present the mechanisms of the antibacterial action of selenium nanoparticles in the form of a list (see below) and in the form of a diagram (Figure 8a).

(1) Degradation of proteins due to the bactericidal action of selenium nanoparticles [112].
(2) Slow emission of selenium ions from the surface of nanoparticles can lead to their interaction with -SH, -NH or -COOH functional groups of proteins and enzymes and the subsequent loss of their tertiary and quaternary structure and functions [125].
(3) SeNPs contribute to the inactivation of the natural mechanisms of membrane transport of ions and nutrients through the cell walls, which blocks the vital activity of the cell [225].
(4) Hyperproduction of ROS, disturbance of membrane potential, and depletion of internal ATP [199].
(5) Inhibition of the activity of the dehydrogenase enzyme, as well as destruction of the integrity of the cell membrane [125].
(6) Inhibition of the ability of bacteria to attach to the surface and form bacterial films [146].
(7) Photocatalytic action against bacteria [226].

Thus, SeNPs can potentially be candidates for antibacterial substitutes and additives against antibiotic-resistant bacteria. Antimicrobial NPs can damage bacterial cells through multiple pathways. This multimodal antimicrobial behavior makes nanoparticles attractive, as bacteria are expected to have difficulty developing resistance to multiple forms of attack [227]. Researchers have also found that the viability of eukaryotic cells is preserved under the effective antibacterial action of selenium NPs [199].

In the case of the bacteriostatic action of selenium nanoparticles, the activity of the dehydrogenase enzyme is inhibited, and the integrity of the cell membrane is destroyed. This effect was observed when using selenium nanoparticles stabilized with arabinogalactan polysaccharide [125]. Researchers suggest possible mechanisms of the antibacterial action of selenium nanoparticles, which are triggered by the contact of the nanoparticle with a living cell.

In particular, this is the hyperproduction of ROS on the surface of nanoparticles, followed by a cascade of the LPO (lipid peroxidase) reaction, damage to cell membranes and organelles, blocking of the transcriptional gene and activation of apoptosis genes, as well as impaired synthesis of a number of cellular proteins and enzymes. In addition, the adhesion of nanoparticles on the cell surface can be accompanied by depolarization of the cell membrane, destruction of its integrity and, subsequently, cell death [125]. In the case of the combined bacteriostatic and bactericidal action of selenium nanoparticles, the ability of bacteria to attach to the surface and form bacterial films is presumably inhibited. This conclusion was made in the study of selenium nanoparticles with a silver shell [146]. For convenience, some mechanisms are shown schematically in Figure 8a,d.

Antifungal mechanisms includes antibiofilm activity [221,228], ROS generation and oxidative stress (with the addition of antifungal drug ketoconazole) [229] and influence on expression of fungicidal drug resistance genes [230] (Figure 8b).

The antiviral action of SeNPs is realized through several mechanisms: disruption of the functioning of viral capside proteins (in particular, hemagglutinin and neuraminidase activities of influenza virus), blocking of the virus-induced activation of the AKT-p52-Caspase3-dependent proapoptotic pathway, inhibition of viral replication in the host cell and enhancement of the action of antiviral drugs [183,184,187] (Figure 8c).

## 5. Methods for Studying the Characteristics of Selenium Nanoparticles

To describe the physicochemical characteristics of selenium NPs, a number of methods are usually used in the analyzed literature. Basically, in all articles, data on morphology, size and elemental composition are given.

Various microscopic methods are used to characterize the morphology of NPs. The most common is transmission electron microscopy (TEM) [231], and rarely atomic force microscopy (AFM) [232], scanning tunnelling microscopy (STM) [233] or scanning electron microscopy (SEM).

To characterize the sizes of NPs, the dynamic light scattering (DLS) method is most often used [234]. The method makes it possible to measure the hydrodynamic radius of nanoparticles, that is, the size of the NPs themselves and their solvate shell. The less-commonly used CPS Disc Centrifuge method allows estimation of the size of nanoparticles; however, with sizes less than 7 nm, the procedure can take several hours. Often, the size distribution of NPs is calculated using photographs or reconstructions obtained using microscopy. Differential centrifugal sedimentation (DCS) [235], particle size mobility scanning (SMPS) [236] and ion occlusion scanning (SIOS) are relatively inexpensive methods for determining particle size based on the Coulter counting principle [237]. The rarely used nanoparticle tracking analysis (NTA) method provides information on the diffusion of nanoparticles and their size [238].

A large number of methods are used to characterize the elemental composition of NPs. It should be noted that in biological applications, there are no NPs consisting of selenium oxide, and this greatly simplifies the task, since for most nanoparticles from other elements, it is necessary to prove the absence or presence of oxides [239]. Selenium oxide is soluble in water. When the surface of a selenium nanoparticle is oxidized, selenium oxide goes into solution, and the surface again consists of selenium atoms. The process continues until the complete dissolution of the selenium NP. To characterize the chemical composition, energy dispersive spectroscopy (EDX) [240] is usually used; this is very convenient since this method is usually integrated into modern transmission electron microscopes. X-ray photoelectron spectroscopy (XPS) is also often used [241]. In addition, the crystal structure of nanoparticles is often studied using the X-ray diffraction (XRD) method [242]. Selenium nanoparticles with impurities and conjugates are usually characterized with absorption spectroscopy in the UV–visible region of the spectrum [243] and Fourier transform IR spectroscopy (FTIR) [244,245].

Sometimes, differential scanning calorimetry and the Brunauer–Emmett–Teller (BET) method [246] are used to characterize NPs; these methods are used to study the surface area and rheological properties of NPs. Modulation interference microscopy (MIM) is used to study the spatial distribution of nanoparticles inside a polymer matrix [247]. The stability of NP colloids in a solvent is studied by measuring the zeta potential [227].

## 6. Cytotoxicity to Eukaryotic Cells

In addition to effective antimicrobial activity, it is also important to determine the safety of selenium nanoparticles for eukaryotic cells. This is a fundamental point that will allow the use of selenium nanoparticles in clinical practice. Zeraatkar et al. proved that selenium nanoparticles do not exhibit cytotoxicity to mouse fibroblasts (3T3 cell line) up to a concentration of 64 μg/mL, while the minimum inhibitory concentration is 4–8 μg/mL for *Pseudomonas aeruginosa* and *Acinetobacter baumannii* bacteria [117]. In another work by Jason Hou et al., it was shown that at concentrations from 2 to 16 μg/mL, no cytotoxicity was observed for osteoblast precursor cells (MC3T3-E1 osteoblast precursor cell line), which is important, while at concentrations of 4 μg/mL and more, the growth of the bacterium *Porphyromonas gingivalis* was inhibited [114].

In a published article [191], using selenium nanoparticles synthesized by a biogenic method, it was found that the inhibitory concentrations (IC50 is the concentration sufficient to inhibit the viability of 50% of cells) of SeNPs were 233.08 and 849.21 µg/mL for normal kidney cells and liver cells, respectively. Thus, normal liver cells showed greater viability to selenium nanoparticles compared with kidney cells. Importantly, the minimum inhibitory concentration of such nanoparticles against bacteria of the genera *Salmonella*, *Klebsiella* and *Escherichia* is 25–200 µg/mL, which is much lower than the concentration of cytotoxicity for the studied normal cell lines.

Importantly, the potential application of selenium nanoparticles lies precisely in their selective cytotoxicity; that is, nanoparticles show effective cytotoxicity for cancer cells and are safe for normal cells. For example, an article [131] was published in which the effective concentration for the antiproliferative activity of HeLa cells is 5.5 µg/mL, while the MIC for *E. coli* and *S. aureus* bacteria is 50 µg/mL. Thus, we can speak of effective inhibition of both the growth of cancerous and bacterial cells.

Moreover, a study was conducted and published in our laboratory that showed that selenium nanoparticles can have a cytoprotective effect on neuroglial cells of the cerebral cortex during ischemia/reoxygenation [48]. At concentrations as low as 3 µg/mL, selenium nanoparticles inhibit the hyperproduction of ROS in cells. The experiments also used a complex of selenium NPs with taxifolin (a flavonoid that lacks the “high dosage effect” that occurs in selenium NPs), while at a concentration of 10 µg/mL, the Se-TAX complex inhibits ROS hyperproduction and does not have toxicity on brain cells [48].

SeNPs are less toxic in vivo than other organic and inorganic (selenite) sources of Se [248]. This fact makes SeNPs attractive for biomedical applications. However, a number of experiments have shown that concentrations of SeNPs above 2 mg/kg can cause Se toxicity in mammals. [249]. Manifestations of toxicity at concentrations below 5–30 mg/kg according to the WOS standard require the substance to be classified as Toxicity class I–II. In addition, SeNPs (5 μM) exhibit acute toxicity to marine unicellular algae, which makes products based on SeNPs potentially hazardous to the environment [250]. SeNPs have comparable or even greater toxicity against microorganisms than metal NPs, for example, CuNPs [251]. The average MIC/MBC CuNPs against different strains of Escherichia coli and Staphylococcus aureus are ~210/235 and ~140/160 μg/mL, respectively [252]. For SeNPs, these values can reach <10 μg/mL and <20 μg/mL for MIC and MBC, respectively [114]. The inhibitory concentration for fungi of CuNPs is 13–22 μg/mL [253], which is comparable to or higher than that of SeNPs [150,209,212]. The toxic concentration of CuNPs for animals is 200 μg/kg, which is significantly higher than that of SiNPs, but lower than that of SiNPs [110,111,249]. CuNPs accumulate in the liver, inhibit CYP450 enzymes, and cause activation of pro-inflammatory reactions through the signaling pathway of NF-κB, MAPK, and STAT5 [254]. Thus, SeNPs have more pronounced antimicrobial properties than CuNPs, but may be more toxic against eukaryotes. These facts require caution in further biomedical developments using SeNPs.

## 7. Biomedical Applications

SeNPs can be used not only as antibacterial agents, but also as therapeutic agents for various pathologies. A detailed review of the biomedical applications of SeNPs can be found in [130,255]. Briefly, potential medical applications of SeNPs include cancer therapy (lung carcinoma, breast, liver, bone or kidney cancer, melanoma, etc.) [156,160,256,257,258,259], protection against poisoning (arsenic, patulin) [260,261], consequences of oxidative stress [157,256], therapy of diabetes mellitus I (oral delivery of insulin, relief of the consequences of hyperglycemia) [262,263,264] and Alzheimer’s disease [265]. In addition to the antimicrobial action, SeNPs have antiparasitic activity, which has been shown against protozoa (*Leishmania*, *Promastigote*, *Giardia duodenalis*, *Toxoplasma*) [266,267,268,269], tapeworms (*Cystic echinococcus*, *Echinococcus granulosus*) [270,271] and roundworms (*Trichinella*) [272].

## 8. Conclusions

Today, the problem of antimicrobial resistance is acute. A potential approach that can solve this problem is the use of selenium nanoparticles for antimicrobial activity against viruses, bacteria and fungi. That is why, in recent years, the interest of the scientific community in this topic has grown significantly. Selenium nanoparticles are especially attractive because of their relatively simple and inexpensive synthesis, as well as their low cytotoxicity for eukaryotic cells. Unfortunately, the chronic cytotoxicity of SeNPs has been little studied; therefore, in the future, it is necessary to carefully and thoroughly investigate this issue. It is expected that the development of scientific research in this area will effectively solve the problem of antimicrobial resistance in the near future. We consider that, in practical terms, it will be especially interesting to develop nanocoatings and nanocomposites with antibacterial properties based on SeNPs. It is probably worth focusing especially on antiviral research due to its potentially high threat. Recently, the number of publications on antiviral properties of SeNPs has been growing.

## Figures and Tables

**Table 1:** Examples of recent patents on SeNPs.

№	Patent ID	Patent Name	Reference
1	US9624237B2	Oridonin functionalized selenium nanoparticles and method of preparation thereof	[73]
2	US8445026B2	Selenium nanoparticles with improved biological effects	[90]
3	US10807920B2	Trichoderma-derived selenium nanoparticle foliar fertilizer for reducing crop fungal diseases and toxic contamination	[75]
4	US9259005B2	Antipathogenic surfaces having selenium nanoclusters	[57]
5	CN111214460A	Folic acid-chitosan-nano-selenium tumor-targeted drug delivery system and preparation method thereof	[67]
6	RU2798268C1	Method of obtaining a veterinary drug based on non-specific immunoglobulins and colloidal particles of selenium for the correction of the immune system	[52]
7	JP2011501977A	Method for producing hydrous tissue paper having antibacterial and antifungal functions	[56]
8	KR101120635B1	Method for cultivating high quality and functional vegetable fruit	[80]
9	US20220339187A1	Protein-bound nano-selenium and preparation method and application method thereof	[91]
10	US10875235B2	Bactericidal surface patterns	[58]

## Data Availability

The raw data supporting the conclusions of this article will be made available by the authors, without undue reservation.

## References

[1] Minasyan H. (2019). Sepsis: Mechanisms of bacterial injury to the patient. Scand. J. Trauma Resusc. Emerg. Med..

[2] Lee C.-R., Lee J.H., Park K.S., Kim Y.B., Jeong B.C., Lee S.H. (2016). Global Dissemination of Carbapenemase-Producing *Klebsiella pneumoniae*: Epidemiology, Genetic Context, Treatment Options, and Detection Methods. Front. Microbiol..

[3] Rozgonyi F., Valenta B., Brátovics A., Csire B. (1967). The sensitivity of “polyresistant” microorganisms to new antibiotics. Changes in the resistance to antibiotics of the more important pathogenic bacteria isolated from clinical specimens during 1962–1965. Orvosi Hetil..

[4] Abraham E.P., Chain E. (1940). An Enzyme from Bacteria able to Destroy Penicillin. Nature.

[5] Kozlov A.V., Gusyakova O.A., Lyamin A.V., Kezko J.L., Khaliulin A.V., Ereshchenko A.A. (2018). Polyresistent microflora in the structure of microorganisms divided from blood of patients of the general hospital. Klin. Lab. Diagn..

[6] Kirk M.D., Pires S.M., Black R.E., Caipo M., Crump J.A., Devleesschauwer B., Döpfer D., Fazil A., Fischer-Walker C.L., Hald T. (2015). World Health Organization Estimates of the Global and Regional Disease Burden of 22 Foodborne Bacterial, Protozoal, and Viral Diseases, 2010: A Data Synthesis. PLoS Med..

[7] Rowe S.Y., Rocourt J.R., Shiferaw B., Kassenborg H.D., Segler S.D., Marcus R., Daily P.J., Hardnett F.P., Slutsker L. (2004). Breast-feeding decreases the risk of sporadic salmonellosis among infants in FoodNet sites. Clin. Infect. Dis..

[8] Batz M.B., Henke E., Kowalcyk B. (2013). Long-term consequences of foodborne infections. Infect. Dis. Clin. N. Am..

[9] Kalyoussef S., Feja K.N. (2014). Foodborne Illnesses. Adv. Pediatr..

[10] Quaglia N.C., Dambrosio A. (2018). *Helicobacter pylori*: A foodborne pathogen?. World J. Gastroenterol..

[11] Posfay-Barbe K.M., Wald E.R. (2009). Listeriosis. Semin. Fetal Neonatal Med..

[12] Kelesidis T., Salhotra A., Fleisher J., Uslan D.Z. (2010). Listeria endocarditis in a patient with psoriatic arthritis on infliximab: Are biologic agents as treatment for inflammatory arthritis increasing the incidence of Listeria infections?. J. Infect..

[13] Chlebicz A., Śliżewska K. (2018). Campylobacteriosis, Salmonellosis, Yersiniosis, and Listeriosis as Zoonotic Foodborne Diseases: A Review. Int. J. Environ. Res. Public Health.

[14] Ahmad M., Khan A.U. (2019). Global economic impact of antibiotic resistance: A review. J. Glob. Antimicrob. Resist..

[15] Gebreyes W.A., Thakur S. (2005). Multidrug-Resistant *Salmonella enterica* Serovar Muenchen from Pigs and Humans and Potential Interserovar Transfer of Antimicrobial Resistance. Antimicrob. Agents Chemother..

[16] Endimiani A., Hujer K.M., Hujer A.M., Bertschy I., Rossano A., Koch C., Gerber V., Francey T., Bonomo R.A., Perreten V. (2011). *Acinetobacter baumannii* isolates from pets and horses in Switzerland: Molecular characterization and clinical data. J. Antimicrob. Chemother..

[17] Fodor A., Varga I., Hevesi M., Mathe-Fodor A., Racsko J., Hogan A.J. (2012). Novel Anti-Microbial Peptides of Xenorhabdus Origin against Multidrug Resistant Plant Pathogens. A Search for Antibacterial Agents.

[18] Ahmed S.A., Barış E., Go D.S., Lofgren H., Osorio-Rodarte I., Thierfelder K. (2018). Assessing the global poverty effects of antimicrobial resistance. World Dev..

[19] Aziz R.K., Bhullar K., Waglechner N., Pawlowski A., Koteva K., Banks E.D., Johnston M.D., Barton H.A., Wright G.D. (2012). Antibiotic Resistance Is Prevalent in an Isolated Cave Microbiome. PLoS ONE.

[20] Blair J.M.A., Richmond G.E., Piddock L.J.V. (2014). Multidrug efflux pumps in Gram-negative bacteria and their role in antibiotic resistance. Future Microbiol..

[21] Cowen L.E., Sanglard D., Howard S.J., Rogers P.D., Perlin D.S. (2015). Mechanisms of Antifungal Drug Resistance. Cold Spring Harb. Perspect. Med..

[22] Brown G.D., Denning D.W., Gow N.A.R., Levitz S.M., Netea M.G., White T.C. (2012). Hidden Killers: Human Fungal Infections. Sci. Transl. Med..

[23] Tian L., Pang Z., Li M., Lou F., An X., Zhu S., Song L., Tong Y., Fan H., Fan J. (2022). Molnupiravir and Its Antiviral Activity against COVID-19. Front. Immunol..

[24] Gudkov S.V., Burmistrov D.E., Serov D.A., Rebezov M.B., Semenova A.A., Lisitsyn A.B. (2021). Do Iron Oxide Nanoparticles Have Significant Antibacterial Properties?. Antibiotics.

[25] Gudkov S.V., Burmistrov D.E., Serov D.A., Rebezov M.B., Semenova A.A., Lisitsyn A.B. (2021). A Mini Review of Antibacterial Properties of ZnO Nanoparticles. Front. Phys..

[26] Gudkov S.V., Burmistrov D.E., Smirnova V.V., Semenova A.A., Lisitsyn A.B. (2022). A Mini Review of Antibacterial Properties of Al_2_O_3_ Nanoparticles. Nanomaterials.

[27] Giedraitienė A., Ruzauskas M., Šiugždinienė R., Tučkutė S., Milcius D. (2022). Antimicrobial Properties of CuO Particles Deposited on a Medical Mask. Materials.

[28] Li R., Mansukhani N.D., Guiney L.M., Ji Z., Zhao Y., Chang C.H., French C.T., Miller J.F., Hersam M.C., Nel A.E. (2016). Identification and Optimization of Carbon Radicals on Hydrated Graphene Oxide for Ubiquitous Antibacterial Coatings. ACS Nano.

[29] Zheng H., Ma R., Gao M., Tian X., Li Y.-Q., Zeng L., Li R. (2018). Antibacterial applications of graphene oxides: Structure-activity relationships, molecular initiating events and biosafety. Sci. Bull..

[30] Zheng H., Ji Z., Roy K.R., Gao M., Pan Y., Cai X., Wang L., Li W., Chang C.H., Kaweeteerawat C. (2019). Engineered Graphene Oxide Nanocomposite Capable of Preventing the Evolution of Antimicrobial Resistance. ACS Nano.

[31] Pan Y., Zheng H., Li G., Li Y., Jiang J., Chen J., Xie Q., Wu D., Ma R., Liu X. (2022). Antibiotic-Like Activity of Atomic Layer Boron Nitride for Combating Resistant Bacteria. ACS Nano.

[32] Xie M., Gao M., Yun Y., Malmsten M., Rotello V.M., Zboril R., Akhavan O., Kraskouski A., Amalraj J., Cai X. (2023). Antibacterial Nanomaterials: Mechanisms, Impacts on Antimicrobial Resistance and Design Principles. Angew. Chem. Int. Ed..

[33] Yougbaré S., Mutalik C., Okoro G., Lin I.H., Krisnawati D.I., Jazidie A., Nuh M., Chang C.-C., Kuo T.-R. (2021). Emerging Trends in Nanomaterials for Antibacterial Applications. Int. J. Nanomed..

[34] Yougbare S., Chang T.-K., Tan S.-H., Kuo J.-C., Hsu P.-H., Su C.-Y., Kuo T.-R. (2019). Antimicrobial Gold Nanoclusters: Recent Developments and Future Perspectives. Int. J. Mol. Sci..

[35] Lin M.-H., Wang Y.-H., Kuo C.-H., Ou S.-F., Huang P.-Z., Song T.-Y., Chen Y.-C., Chen S.-T., Wu C.-H., Hsueh Y.-H. (2021). Hybrid ZnO/chitosan antimicrobial coatings with enhanced mechanical and bioactive properties for titanium implants. Carbohydr. Polym..

[36] Butler K.S., Peeler D.J., Casey B.J., Dair B.J., Elespuru R.K. (2015). Silver nanoparticles: Correlating nanoparticle size and cellular uptake with genotoxicity. Mutagenesis.

[37] Shuguang W., Lawson R., Ray P.C., Hongtao Y. (2011). Toxic effects of gold nanoparticles on *Salmonella typhimurium* bacteria. Toxicol. Ind. Health.

[38] Panáček A., Kvítek L., Smékalová M., Večeřová R., Kolář M., Röderová M., Dyčka F., Šebela M., Prucek R., Tomanec O. (2017). Bacterial resistance to silver nanoparticles and how to overcome it. Nat. Nanotechnol..

[39] Li X.Z., Nikaido H., Williams K.E. (1997). Silver-resistant mutants of *Escherichia coli* display active efflux of Ag+ and are deficient in porins. J. Bacteriol..

[40] Niño-Martínez N., Salas Orozco M.F., Martínez-Castañón G.-A., Torres Méndez F., Ruiz F. (2019). Molecular Mechanisms of Bacterial Resistance to Metal and Metal Oxide Nanoparticles. Int. J. Mol. Sci..

[41] Amaro F., Morón Á., Díaz S., Martín-González A., Gutiérrez J.C. (2021). Metallic Nanoparticles-Friends or Foes in the Battle against Antibiotic-Resistant Bacteria?. Microorganisms.

[42] Helmlinger J., Sengstock C., Groß-Heitfeld C., Mayer C., Schildhauer T., Köller M., Epple M. (2016). Silver nanoparticles with different size and shape: Equal cytotoxicity, but different antibacterial effects. RSC Adv..

[43] Napierska D., Thomassen L.C.J., Lison D., Martens J.A., Hoet P.H. (2010). The nanosilica hazard: Another variable entity. Part. Fibre Toxicol..

[44] Guo C., Wang J., Jing L., Ma R., Liu X., Gao L., Cao L., Duan J., Zhou X., Li Y. (2018). Mitochondrial dysfunction, perturbations of mitochondrial dynamics and biogenesis involved in endothelial injury induced by silica nanoparticles. Environ. Pollut..

[45] Farooq A., Whitehead D., Azzawi M. (2014). Attenuation of endothelial-dependent vasodilator responses, induced by dye-encapsulated silica nanoparticles, in aortic vessels. Nanomedicine.

[46] Maltseva V.N., Gudkov S., Turovsky E. (2022). Modulation of the Functional State of Mouse Neutrophils by Selenium Nanoparticles In Vivo. Int. J. Mol. Sci..

[47] Varlamova E., Plotnikov E., Gudkov S., Turovsky E. (2022). Size-Dependent Cytoprotective Effects of Selenium Nanoparticles during Oxygen-Glucose Deprivation in Brain Cortical Cells. Int. J. Mol. Sci..

[48] Varlamova E.G., Khabatova V.V., Gudkov S.V., Plotnikov E.Y., Turovsky E.A. (2022). Cytoprotective Properties of a New Nanocomplex of Selenium with Taxifolin in the Cells of the Cerebral Cortex Exposed to Ischemia/Reoxygenation. Pharmaceutics.

[49] Varlamova E., Goltyaev M., Simakin A., Gudkov S., Turovsky E. (2022). Comparative Analysis of the Cytotoxic Effect of a Complex of Selenium Nanoparticles Doped with Sorafenib, “Naked” Selenium Nanoparticles, and Sorafenib on Human Hepatocyte Carcinoma HepG2 Cells. Int. J. Mol. Sci..

[50] Malyugina S., Skalickova S., Skladanka J., Slama P., Horky P. (2021). Biogenic Selenium Nanoparticles in Animal Nutrition: A Review. Agriculture.

[51] Zhang J., Wang X., Xu T. (2008). Elemental Selenium at Nano Size (Nano-Se) as a Potential Chemopreventive Agent with Reduced Risk of Selenium Toxicity: Comparison with Se-Methylselenocysteine in Mice. Toxicol. Sci..

[52] Kozlov S.V., Staroverov S.A., Skvortsova N.I., Soldatov D.A., Chekunov M.A., Silina E.V., Kozlov E.S., Artemev D.A. (2023). Method of Obtaining a Veterinary Drug Based on Non-Specific Immunoglobulins and Colloidal Particles of Selenium for the Correction of the Immune System. RU Patent.

[53] Chen C., Hu H., Li X., Zheng Z., Wang Z., Wang X., Zheng P., Cui F., Li G., Wang Y. (2022). Rapid Detection of Anti-SARS-CoV-2 Antibody Using a Selenium Nanoparticle-Based Lateral Flow Immunoassay. IEEE Trans. NanoBiosci..

[54] Wang Z., Zheng Z., Hu H., Zhou Q., Liu W., Li X., Liu Z., Wang Y., Ma Y. (2020). A point-of-care selenium nanoparticle-based test for the combined detection of anti-SARS-CoV-2 IgM and IgG in human serum and blood. Lab A Chip.

[55] Wang Q., Webster T.J. (2012). Nanostructured selenium for preventing biofilm formation on polycarbonate medical devices. J. Biomed. Mater. Res. Part A.

[56] Wa N.J. (2007). Method for Producing Hydrous Tissue Paper Having Antibacterial and Antifungal Functions. JP Patent.

[57] Webster T.J., Tran P.A. (2016). Antipathogenic Surfaces Having Selenium Nanoclusters. U.S. Patent.

[58] Yeee A.F., Liang L., Ing N., Gibbs M., Dickson M.N. (2020). Bactericidal Surface Patterns. U.S. Patent.

[59] Fang M., Zhang H., Wang Y., Zhang H., Zhang D., Xu P. (2023). Biomimetic selenium nanosystems for infectious wound healing. Eng. Regen..

[60] Abbaszadeh A., Tehmasebi-Foolad A., Rajabzadeh A., Beigi-Brojeni N., Zarei L. (2019). Effects of Chitosan/Nano Selenium Biofilm on Infected Wound Healing in Rats; An Experimental Study. Bull. Emerg. Trauma.

[61] Huang W., Hu B., Yuan Y., Fang H., Jiang J., Li Q., Zhuo Y., Yang X., Wei J., Wang X. (2023). Visible Light-Responsive Selenium Nanoparticles Combined with Sonodynamic Therapy to Promote Wound Healing. ACS Biomater. Sci. Eng..

[62] Maiyo F., Singh M. (2017). Selenium nanoparticles: Potential in cancer gene and drug delivery. Nanomedicine.

[63] Dipak N., Loveleen K., Harvinder S.S., Dharambeer M.S., Sonali G., Deepa T. (2022). Application of Selenium Nanoparticles in Localized Drug Targeting for Cancer Therapy. Anti-Cancer Agents Med. Chem..

[64] Wu H., Li X., Liu W., Chen T., Li Y., Zheng W., Man C.W.-Y., Wong M.-K., Wong K.-H. (2012). Surface decoration of selenium nanoparticles by mushroom polysaccharides–protein complexes to achieve enhanced cellular uptake and antiproliferative activity. J. Mater. Chem..

[65] Tang S., Wang T., Jiang M., Huang C., Lai C., Fan Y., Yong Q. (2019). Construction of arabinogalactans/selenium nanoparticles composites for enhancement of the antitumor activity. Int. J. Biol. Macromol..

[66] Sun D., Liu Y., Yu Q., Qin X., Yang L., Zhou Y., Chen L., Liu J. (2014). Inhibition of tumor growth and vasculature and fluorescence imaging using functionalized ruthenium-thiol protected selenium nanoparticles. Biomaterials.

[67] Dong F., Zhang L., Li R., Feng F., Wang W., Li D., Xiang Q., Yan P. (2020). Folic Acid-Chitosan-Nano-Selenium Tumor Targeted Drug Delivery System and Preparation Method Thereof. CN Patent.

[68] Xia Y., Zhao M., Chen Y., Hua L., Xu T., Wang C., Li Y., Zhu B. (2018). Folate-targeted selenium nanoparticles deliver therapeutic siRNA to improve hepatocellular carcinoma therapy. RSC Adv..

[69] Zou J., Su S., Chen Z., Liang F., Zeng Y., Cen W., Zhang X., Xia Y., Huang D. (2019). Hyaluronic acid-modified selenium nanoparticles for enhancing the therapeutic efficacy of paclitaxel in lung cancer therapy. Artif. Cells Nanomed. Biotechnol..

[70] Zhang Y., Li X., Huang Z., Zheng W., Fan C., Chen T. (2013). Enhancement of cell permeabilization apoptosis-inducing activity of selenium nanoparticles by ATP surface decoration. Nanomed. Nanotechnol. Biol. Med..

[71] Goltyaev M.V., Varlamova E.G. (2023). The Role of Selenium Nanoparticles in the Treatment of Liver Pathologies of Various Natures. Int. J. Mol. Sci..

[72] Varlamova E.G., Khabatova V.V., Gudkov S.V., Turovsky E.A. (2023). Ca^2+^-Dependent Effects of the Selenium-Sorafenib Nanocomplex on Glioblastoma Cells and Astrocytes of the Cerebral Cortex: Anticancer Agent and Cytoprotector. Int. J. Mol. Sci..

[73] Jiang P.I., Cai J., Hua J. (2017). Oridonin Functionalized Nanoparticles and Method of Preparation Thereof Selenium. U.S. Patent.

[74] Walsh S.K., Kamali N., McGrath J., Hogan J.J., Hanrahan J.P. (2023). Multidimensional Application of Selenium Nanoparticles. https://glantreo.com/multidimensional-application-of-selenium-nanoparticles/.

[75] Wu A., Hu D., Na L.I.U., Yu S., Yu D., Tang Y., Wang Y. (2020). Trichoderma-Derived Selenium Nanoparticles Foliar Fertilizer for Reducing Crop Fungal Diseases and Toxin Contamination. U.S. Patent.

[76] Usmani Z., Kumar A., Tripti, Ahirwal J., Prasad M.N.V., Prasad M.N.V. (2019). Chapter 20—Scope for Applying Transgenic Plant Technology for Remediation and Fortification of Selenium. Transgenic Plant Technology for Remediation of Toxic Metals and Metalloids.

[77] Wang Q., Yu Y., Li J., Wan Y., Huang Q., Guo Y., Li H. (2017). Effects of Different Forms of Selenium Fertilizers on Se Accumulation, Distribution, and Residual Effect in Winter Wheat–Summer Maize Rotation System. J. Agric. Food Chem..

[78] Gudkov S.V., Shafeev G.A., Glinushkin A.P., Shkirin A.V., Barmina E.V., Rakov I.I., Simakin A.V., Kislov A.V., Astashev M.E., Vodeneev V.A. (2020). Production and Use of Selenium Nanoparticles as Fertilizers. ACS Omega.

[79] Shafeev G., Barmina E., Valiullin L., Simakin A., Ovsyankina A., Demin D., Kosolapov V., Korshunov A., Denisov R. (2019). Soil fertilizer based on selenium nanoparticles. IOP Conf. Ser. Earth Environ. Sci..

[80] Hak K., Jong L., Hyo K., Gwang L., Jun P., Chan L., St Y. (2012). Method for Cultivating High Quality and Functional Vegetable Fruit. KR Patent.

[81] Fouda A., Al-Otaibi W.A., Saber T., AlMotwaa S.M., Alshallash K.S., Elhady M., Badr N.F., Abdel-Rahman M.A. (2022). Antimicrobial, Antiviral, and In-Vitro Cytotoxicity and Mosquitocidal Activities of Portulaca oleracea-Based Green Synthesis of Selenium Nanoparticles. J. Funct. Biomater..

[82] Ahmed F., Dwivedi S., Shaalan N.M., Kumar S., Arshi N., Alshoaibi A., Husain F.M. (2020). Development of Selenium Nanoparticle Based Agriculture Sensor for Heavy Metal Toxicity Detection. Agriculture.

[83] Dumore N.S., Mukhopadhyay M. (2020). Sensitivity enhanced SeNPs-FTO electrochemical sensor for hydrogen peroxide detection. J. Electroanal. Chem..

[84] Mostafavi E., Medina-Cruz D., Truong L.B., Kaushik A., Iravani S. (2022). Selenium-based nanomaterials for biosensing applications. Mater. Adv..

[85] Amani H., Habibey R., Shokri F., Hajmiresmail S.J., Akhavan O., Mashaghi A., Pazoki-Toroudi H. (2019). Selenium nanoparticles for targeted stroke therapy through modulation of inflammatory and metabolic signaling. Sci. Rep..

[86] El-Ghazaly M.A., Fadel N., Rashed E., El-Batal A., Kenawy S.A. (2017). Anti-inflammatory effect of selenium nanoparticles on the inflammation induced in irradiated rats. Can. J. Physiol. Pharmacol..

[87] Gao X., Zhang J., Zhang L. (2002). Hollow Sphere Selenium Nanoparticles: Their In-Vitro Anti Hydroxyl Radical Effect. Adv. Mater..

[88] Torres S.K., Campos V.L., León C.G., Rodríguez-Llamazares S.M., Rojas S.M., González M., Smith C., Mondaca M.A. (2012). Biosynthesis of selenium nanoparticles by *Pantoea agglomerans* and their antioxidant activity. J. Nanoparticle Res..

[89] Ahmed H.H., Abd El-Maksoud M.D., Abdel Moneim A.E., Aglan H.A. (2016). Pre-Clinical Study for the Antidiabetic Potential of Selenium Nanoparticles. Biol. Trace Elem. Res..

[90] Gao X., Sun Y. (2013). Selenium Nanoparticles with Improved Biological Effects. U.S. Patent.

[91] Peng Y., Peng L., Liu T. (2022). Protein-Bound Nano-Selenium and Preparation Method and Application Thereof. CN Patent.

[92] Selvarajan V., Obuobi S., Ee P.L.R. (2020). Silica Nanoparticles—A Versatile Tool for the Treatment of Bacterial Infections. Front. Chem..

[93] Jafari S., Derakhshankhah H., Alaei L., Fattahi A., Varnamkhasti B.S., Saboury A.A. (2019). Mesoporous silica nanoparticles for therapeutic/diagnostic applications. Biomed. Pharmacother..

[94] Stöber W., Fink A., Bohn E. (1968). Controlled growth of monodisperse silica spheres in the micron size range. J. Colloid Interface Sci..

[95] Zulfiqar U., Subhani T., Wilayat Husain S. (2016). Synthesis of silica nanoparticles from sodium silicate under alkaline conditions. J. Sol-Gel Sci. Technol..

[96] Kuddus A., Islam R., Tabassum S., Ismail A.B. (2019). Synthesis of Si NPs from River Sand Using the Mechanochemical Process and its Applications in Metal Oxide Heterojunction Solar Cells. Silicon.

[97] Zhou Y., Quan G., Wu Q., Zhang X., Niu B., Wu B., Huang Y., Pan X., Wu C. (2018). Mesoporous silica nanoparticles for drug and gene delivery. Acta Pharm. Sin. B.

[98] Zhang Y., Wang J., Bai X., Jiang T., Zhang Q., Wang S. (2012). Mesoporous Silica Nanoparticles for Increasing the Oral Bioavailability and Permeation of Poorly Water Soluble Drugs. Mol. Pharm..

[99] Makarovsky I., Boguslavsky Y., Alesker M., Lellouche J., Banin E., Lellouche J.-P. (2011). Novel Triclosan-Bound Hybrid-Silica Nanoparticles and their Enhanced Antimicrobial Properties. Adv. Funct. Mater..

[100] Wu S.-H., Lin Y.-S., Hung Y., Chou Y.-H., Hsu Y.-H., Chang C., Mou C.-Y. (2008). Multifunctional Mesoporous Silica Nanoparticles for Intracellular Labeling and Animal Magnetic Resonance Imaging Studies. ChemBioChem.

[101] Pandey S., Mewada A., Thakur M., Pillai S., Dharmatti R., Phadke C., Sharon M. (2014). Synthesis of mesoporous silica oxide/C-dot complex (meso-SiO2/C-dots) using pyrolysed rice husk and its application in bioimaging. RSC Adv..

[102] Li Z., Zhang Y., Wu X., Wu X., Maudgal R., Zhang H., Han G. (2015). In Vivo Repeatedly Charging Near-Infrared-Emitting Mesoporous SiO_2_/ZnGa_2_O_4_:Cr^3+^ Persistent Luminescence Nanocomposites. Adv. Sci..

[103] Chen Z., Tan Y., Xu K., Zhang L., Qiu B., Guo L., Lin Z., Chen G. (2016). Stimulus-response mesoporous silica nanoparticle-based chemiluminescence biosensor for cocaine determination. Biosens. Bioelectron..

[104] Bai Y., Yang H., Yang W., Li Y., Sun C. (2007). Gold nanoparticles-mesoporous silica composite used as an enzyme immobilization matrix for amperometric glucose biosensor construction. Sens. Actuators B Chem..

[105] Moon J.H., McDaniel W., Hancock L.F. (2006). Facile fabrication of poly(p-phenylene ethynylene)/colloidal silica composite for nucleic acid detection. J. Colloid Interface Sci..

[106] Boora R., Sheoran P., Rani N., Kumari S., Thakur R., Grewal S. (2023). Biosynthesized Silica Nanoparticles (Si NPs) Helps in Mitigating Drought Stress in Wheat Through Physiological Changes and Upregulation of Stress Genes. Silicon.

[107] Li L.-L., Wang H. (2013). Enzyme-Coated Mesoporous Silica Nanoparticles as Efficient Antibacterial Agents In Vivo. Adv. Healthc. Mater..

[108] González B., Colilla M., Díez J., Pedraza D., Guembe M., Izquierdo-Barba I., Vallet-Regí M. (2018). Mesoporous silica nanoparticles decorated with polycationic dendrimers for infection treatment. Acta Biomater..

[109] Díaz-García D., Ardiles P.R., Díaz-Sánchez M., Mena-Palomo I., del Hierro I., Prashar S., Rodríguez-Diéguez A., Páez P.L., Gómez-Ruiz S. (2020). Copper-functionalized nanostructured silica-based systems: Study of the antimicrobial applications and ROS generation against gram positive and gram negative bacteria. J. Inorg. Biochem..

[110] Kim M., Park J.-H., Jeong H., Hong J., Choi W.S., Lee B.-H., Park C.Y. (2017). An Evaluation of the in vivo Safety of Nonporous Silica Nanoparticles: Ocular Topical Administration versus Oral Administration. Sci. Rep..

[111] An S.S.A., Ryu H.J., Seong N.-W., So B.J., Seo H.-S., Kim J.-H., Hong J.-S., Park M.-K., Kim M.-S., Kim Y.-R. (2014). Evaluation of silica nanoparticle toxicity after topical exposure for 90 days. Int. J. Nanomed..

[112] Han H.-W., Patel K.D., Kwak J.-H., Jun S.-K., Jang T.-S., Lee S.-H., Knowles J.C., Kim H.-W., Lee H.-H., Lee J.-H. (2021). Selenium Nanoparticles as Candidates for Antibacterial Substitutes and Supplements against Multidrug-Resistant Bacteria. Biomolecules.

[113] Geoffrion L.D., Hesabizadeh T., Medina-Cruz D., Kusper M., Taylor P., Vernet-Crua A., Chen J., Ajo A., Webster T.J., Guisbiers G. (2020). Naked Selenium Nanoparticles for Antibacterial and Anticancer Treatments. ACS Omega.

[114] Hou J., Tamura Y., Lu H.-Y., Takahashi Y., Kasugai S., Nakata H., Kuroda S. (2022). An In Vitro Evaluation of Selenium Nanoparticles on Osteoblastic Differentiation and Antimicrobial Properties against *Porphyromonas gingivalis*. Nanomaterials.

[115] Zhang L., Li Z., Zhang L., Lei Z., Jin L., Cao J., Quan C. (2022). High-Efficiency Reducing Strain for Producing Selenium Nanoparticles Isolated from Marine Sediment. Int. J. Mol. Sci..

[116] Afzal B., Yasin D., Naaz, Sami N., Zaki A., Kumar R., Srivastava P., Fatma T. (2021). Biomedical potential of *Anabaena variabilis* NCCU-44 based Selenium nanoparticles and their comparison with commercial SeNPs. Sci. Rep..

[117] Zeraatkar S., Tahan M., Sadeghian H., Nazari R., Behmadi M., Hosseini Bafghi M. (2023). Effect of biosynthesized selenium nanoparticles using Nepeta extract against multidrug-resistant *Pseudomonas aeruginosa* and *Acinetobacter baumannii*. J. Basic Microbiol..

[118] Lin W., Zhang J., Xu J.-F., Pi J. (2021). The Advancing of Selenium Nanoparticles Against Infectious Diseases. Front. Pharmacol..

[119] Martínez-Esquivias F., Guzmán-Flores J.M., Pérez-Larios A., González Silva N., Becerra-Ruiz J.S. (2021). A Review of the Antimicrobial Activity of Selenium Nanoparticles. J. Nanosci. Nanotechnol..

[120] Kopel J., Fralick J., Reid T.W. (2022). The Potential Antiviral Effects of Selenium Nanoparticles and Coated Surfaces. Antibiotics.

[121] Vahdati M., Tohidi Moghadam T. (2020). Synthesis and Characterization of Selenium Nanoparticles-Lysozyme Nanohybrid System with Synergistic Antibacterial Properties. Sci. Rep..

[122] Abou Elmaaty T., Sayed-Ahmed K., Mohamed Ali R., El-Khodary K., Abdeldayem S.A. (2022). Simultaneous Sonochemical Coloration and Antibacterial Functionalization of Leather with Selenium Nanoparticles (SeNPs). Polymers.

[123] Kora A.J. (2018). Tree gum stabilised selenium nanoparticles: Characterisation and antioxidant activity. IET Nanobiotechnol..

[124] Dang-Bao T., Ho T.G.-T., Do B.L., Phung Anh N., Phan T.D.T., Tran T.B.Y., Duong N.L., Hong Phuong P., Nguyen T. (2022). Green Orange Peel-Mediated Bioinspired Synthesis of Nanoselenium and Its Antibacterial Activity against Methicillin-Resistant *Staphylococcus aureus*. ACS Omega.

[125] Lesnichaya M., Perfileva A., Nozhkina O., Gazizova A., Graskova I. (2022). Synthesis, toxicity evaluation and determination of possible mechanisms of antimicrobial effect of arabinogalactane-capped selenium nanoparticles. J. Trace Elem. Med. Biol..

[126] Hosseini Bafghi M., Darroudi M., Zargar M., Zarrinfar H., Nazari R. (2021). Biosynthesis of selenium nanoparticles by *Aspergillus flavus* and *Candida albicans* for antifungal applications. Micro Nano Lett..

[127] Islam S.N., Naqvi S.M.A., Raza A., Jaiswal A., Singh A.K., Dixit M., Barnwal A., Gambhir S., Ahmad A. (2022). Mycosynthesis of highly fluorescent selenium nanoparticles from *Fusarium oxysporum*, their antifungal activity against black fungus *Aspergillus niger*, and in-vivo biodistribution studies. 3 Biotech.

[128] Jadhav A.A., Khanna P.K. (2015). Impact of microwave irradiation on cyclo-octeno-1,2,3-selenadiazole: Formation of selenium nanoparticles and their polymorphs. RSC Adv..

[129] Varlamova E.G., Turovsky E.A., Blinova E.V. (2021). Therapeutic Potential and Main Methods of Obtaining Selenium Nanoparticles. Int. J. Mol. Sci..

[130] Khanna P., Bisht N., Singh P. (2022). Selenium Nanoparticles: A Review on Synthesis and Biomedical Applications. Mater. Adv..

[131] Pandey S., Awasthee N., Shekher A., Rai L.C., Gupta S.C., Dubey S.K. (2021). Biogenic synthesis and characterization of selenium nanoparticles and their applications with special reference to antibacterial, antioxidant, anticancer and photocatalytic activity. Bioprocess Biosyst. Eng..

[132] Salem S.S., Fouda M.M.G., Fouda A., Awad M.A., Al-Olayan E.M., Allam A.A., Shaheen T.I. (2021). Antibacterial, Cytotoxicity and Larvicidal Activity of Green Synthesized Selenium Nanoparticles Using *Penicillium corylophilum*. J. Clust. Sci..

[133] Kis-Csitári J., Kónya Z., Kiricsi I. (2008). Sonochemical Synthesis of Inorganic Nanoparticles. Functionalized Nanoscale Materials, Devices and Systems.

[134] Shar A.H., Lakhan M.N., Wang J., Ahmed M., Alali K.T., Ahmed R., Ali I., Dayo A.Q. (2019). Facile synthesis and characterization of selenium nanoparticles by the hydrothermal approach. Dig. J. Nanomater. Biostruct..

[135] Aditha S.K., Kurdekar A.D., Chunduri L.A.A., Patnaik S., Kamisetti V. (2016). Aqueous based reflux method for green synthesis of nanostructures: Application in CZTS synthesis. MethodsX.

[136] Alhawiti A. (2022). Citric acid-mediated green synthesis of selenium nanoparticles: Antioxidant, antimicrobial, and anticoagulant potential applications. Biomass Convers. Biorefinery.

[137] Mellinas C., Jiménez A., Garrigós M.D.C. (2019). Microwave-Assisted Green Synthesis and Antioxidant Activity of Selenium Nanoparticles Using *Theobroma cacao* L. Bean Shell Extract. Molecules.

[138] Hien N.Q., Tuan P.D., Phu D.V., Quoc L.A., Lan N.T.K., Duy N.N., Hoa T.T. (2018). Gamma Co-60 ray irradiation synthesis of dextran stabilized selenium nanoparticles and their antioxidant activity. Mater. Chem. Phys..

[139] Clifford D.M., Castano C.E., Rojas J.V. (2017). Supported transition metal nanomaterials: Nanocomposites synthesized by ionizing radiation. Radiat. Phys. Chem..

[140] Amin B.H., Ahmed H.Y., El Gazzar E.M., Badawy M.M.M. (2021). Enhancement the Mycosynthesis of Selenium Nanoparticles by Using Gamma Radiation. Dose-Response.

[141] Mosallam F.M., El-Sayyad G.S., Fathy R.M., El-Batal A.I. (2018). Biomolecules-mediated synthesis of selenium nanoparticles using *Aspergillus oryzae* fermented Lupin extract and gamma radiation for hindering the growth of some multidrug-resistant bacteria and pathogenic fungi. Microb. Pathog..

[142] Ayyyzhy K., Voronov V., Gudkov S., Rakov I., Simakin A., Shafeev G. (2019). Laser Fabrication and Fragmentation of Selenium Nanoparticles in Aqueous Media. Phys. Wave Phenom..

[143] Shafeev G.A., Barmina E.V., Pimpha N., Rakov I.I., Simakin A.V., Sharapov M.G., Uvarov O.V., Gudkov S.V. (2021). Laser generation and fragmentation of selenium nanoparticles in water and their testing as an additive to fertilisers. Quantum Electron..

[144] Vasileiadis T., Dracopoulos V., Kollia M., Sygellou L., Yannopoulos S.N. (2019). Synthesis of t-Te and a-Se nanospheres using continuous wave visible light. J. Nanoparticle Res..

[145] Vorozhtsov A., Goncharova D., Gavrilenko E., Nemoykina A., Svetlichnyi V. (2018). Antibacterial activity of zinc oxide nanoparticles obtained by pulsed laser ablation in water and air. MATEC Web Conf..

[146] Liang X., Zhang S., Gadd G.M., McGrath J., Rooney D.W., Zhao Q. (2022). Fungal-derived selenium nanoparticles and their potential applications in electroless silver coatings for preventing pin-tract infections. Regen. Biomater..

[147] Fouda A., Hassan S., Eid A., Abdel-Rahman M., Hamza M. (2022). Light enhanced the antimicrobial, anticancer, and catalytic activities of selenium nanoparticles fabricated by endophytic fungal strain, *Penicillium crustosum* EP-1. Sci. Rep..

[148] Mariadoss A.V.A., Saravanakumar K., Sathiyaseelan A., Naveen K.V., Wang M.-H. (2022). Enhancement of anti-bacterial potential of green synthesized selenium nanoparticles by starch encapsulation. Microb. Pathog..

[149] Hashem A.H., Khalil A.M.A., Reyad A.M., Salem S.S. (2021). Biomedical Applications of Mycosynthesized Selenium Nanoparticles Using *Penicillium expansum* ATTC 36200. Biol. Trace Elem. Res..

[150] Hashem A.H., Salem S.S. (2022). Green and ecofriendly biosynthesis of selenium nanoparticles using Urtica dioica (stinging nettle) leaf extract: Antimicrobial and anticancer activity. Biotechnol. J..

[151] Souza L.M.d.S., Dibo M., Sarmiento J.J.P., Seabra A.B., Medeiros L.P., Lourenço I.M., Kobayashi R.K.T., Nakazato G. (2022). Biosynthesis of selenium nanoparticles using combinations of plant extracts and their antibacterial activity. Curr. Res. Green Sustain. Chem..

[152] Shah V., Medina-Cruz D., Vernet-Crua A., Truong L.B., Sotelo E., Mostafavi E., González M.U., García-Martín J.M., Cholula-Díaz J.L., Webster T.J. (2023). Pepper-Mediated Green Synthesis of Selenium and Tellurium Nanoparticles with Antibacterial and Anticancer Potential. J. Funct. Biomater..

[153] Nikam P.B., Salunkhe J.D., Minkina T., Rajput V.D., Kim B.S., Patil S.V. (2022). A review on green synthesis and recent applications of red nano Selenium. Results Chem..

[154] ElSaied B.E.F., Diab A.M., Tayel A.A., Alghuthaymi M.A., Moussa S.H. (2021). Potent antibacterial action of phycosynthesized selenium nanoparticles using *Spirulina platensis* extract. Green Process. Synth..

[155] Mulla N.A., Otari S.V., Bohara R.A., Yadav H.M., Pawar S.H. (2020). Rapid and size-controlled biosynthesis of cytocompatible selenium nanoparticles by Azadirachta indica leaves extract for antibacterial activity. Mater. Lett..

[156] Pansare A.V., Kulal D.K., Shedge A.A., Patil V.R. (2016). hsDNA groove binding, photocatalytic activity, and in vitro breast and colon cancer cell reducing function of greener SeNPs. Dalton Trans..

[157] Vyas J., Rana S. (2017). Antioxidant activity and green synthesis of selenium nanoparticles using allium sativum extract. Int. J. Phytomed..

[158] Xu C., Qiao L., Guo Y., Ma L., Cheng Y. (2018). Preparation, characteristics and antioxidant activity of polysaccharides and proteins-capped selenium nanoparticles synthesized by *Lactobacillus casei* ATCC 393. Carbohydr. Polym..

[159] Khiralla G.M., El-Deeb B.A. (2015). Antimicrobial and antibiofilm effects of selenium nanoparticles on some foodborne pathogens. LWT-Food Sci. Technol..

[160] Ali E.N., El-Sonbaty S.M., Salem F.M. (2013). Evaluation of selenium nanoparticles as a potential chemopreventive agent against lung carcinoma. Int. J. Pharmacol. Biol. Sci..

[161] Zhang W., Chen Z., Liu H., Zhang L., Gao P., Li D. (2011). Biosynthesis and structural characteristics of selenium nanoparticles by Pseudomonas alcaliphila. Colloids Surf. B Biointerfaces.

[162] Yazdi M.H., Mahdavi M., Faghfuri E., Faramarzi M.A., Sepehrizadeh Z., Mohammad Hassan Z., Gholami M., Shahverdi A.R. (2015). Th1 Immune Response Induction by Biogenic Selenium Nanoparticles in Mice with Breast Cancer: Preliminary Vaccine Model. Iran. J. Biotechnol..

[163] Zhang H., Zhou H., Bai J., Li Y., Yang J., Ma Q., Qu Y. (2019). Biosynthesis of selenium nanoparticles mediated by fungus *Mariannaea* sp. HJ and their characterization. Colloids Surf. A Physicochem. Eng. Asp..

[164] Hariharan N., Al-Harbi P., Karuppiah S.R. (2012). Microbial synthesis of selenium nanocomposite using *Saccharomyces cerevisiae* and its antimicrobial activity against pathogens causing nosocomial infection. Chalcogenide Lett..

[165] Lian S., Diko C.S., Yan Y., Li Z., Zhang H., Ma Q., Qu Y. (2019). Characterization of biogenic selenium nanoparticles derived from cell-free extracts of a novel yeast *Magnusiomyces ingens*. 3 Biotech.

[166] Song X., Chen Y., Sun H., Liu X., Leng X. (2020). Physicochemical and functional properties of chitosan-stabilized selenium nanoparticles under different processing treatments. Food Chem..

[167] Ndwandwe B.K., Malinga S.P., Kayitesi E., Dlamini B.C. (2021). Solvothermal synthesis of selenium nanoparticles with polygonal-like nanostructure and antibacterial potential. Mater. Lett..

[168] Youssef D.M., Alshubaily F.A., Tayel A.A., Alghuthaymi M.A., Al-Saman M.A. (2022). Application of Nanocomposites from Bees Products and Nano-Selenium in Edible Coating for Catfish Fillets Biopreservation. Polymers.

[169] Salem S.S., Badawy M.S.E.M., Al-Askar A.A., Arishi A.A., Elkady F.M., Hashem A.H. (2022). Green Biosynthesis of Selenium Nanoparticles Using Orange Peel Waste: Characterization, Antibacterial and Antibiofilm Activities against Multidrug-Resistant Bacteria. Life.

[170] Niranjan R., Zafar S., Lochab B., Priyadarshini R. (2022). Synthesis and Characterization of Sulfur and Sulfur-Selenium Nanoparticles Loaded on Reduced Graphene Oxide and Their Antibacterial Activity against Gram-Positive Pathogens. Nanomaterials.

[171] Dorazilová J., Muchová J., Šmerková K., Kočiová S., Diviš P., Kopel P., Veselý R., Pavliňáková V., Adam V., Vojtová L. (2020). Synergistic Effect of Chitosan and Selenium Nanoparticles on Biodegradation and Antibacterial Properties of Collagenous Scaffolds Designed for Infected Burn Wounds. Nanomaterials.

[172] Muchová J., Hearnden V., Michlovská L., Vištejnová L., Zavaďáková A., Šmerková K., Kočiová S., Adam V., Kopel P., Vojtová L. (2021). Mutual influence of selenium nanoparticles and FGF2-STAB® on biocompatible properties of collagen/chitosan 3D scaffolds: In vitro and ex ovo evaluation. J. Nanobiotechnol..

[173] Huang T., Holden J.A., Reynolds E.C., Heath D.E., O’Brien-Simpson N.M., O’Connor A.J. (2020). Multifunctional Antimicrobial Polypeptide-Selenium Nanoparticles Combat Drug-Resistant Bacteria. ACS Appl. Mater. Interfaces.

[174] Abou Elmaaty T., Sayed-Ahmed K., Elsisi H., Ramadan S.M., Sorour H., Magdi M., Abdeldayem S.A. (2022). Novel Antiviral and Antibacterial Durable Polyester Fabrics Printed with Selenium Nanoparticles (SeNPs). Polymers.

[175] Hussein H.G., El-Sayed E.-S.R., Younis N.A., Hamdy A.E.H.A., Easa S.M. (2022). Harnessing endophytic fungi for biosynthesis of selenium nanoparticles and exploring their bioactivities. AMB Express.

[176] Bilek O., Fohlerová Z., Hubalek J. (2019). Enhanced antibacterial and anticancer properties of Se-NPs decorated TiO_2_ nanotube film. PLoS ONE.

[177] Staats K., Pilz M., Sun J., Boiadjieva-Scherzer T., Kronberger H., Tobudic S., Windhager R., Holinka J. (2022). Antimicrobial potential and osteoblastic cell growth on electrochemically modified titanium surfaces with nanotubes and selenium or silver incorporation. Sci. Rep..

[178] Liu W., Golshan N.H., Deng X., Hickey D.J., Zeimer K., Li H., Webster T.J. (2016). Selenium nanoparticles incorporated into titania nanotubes inhibit bacterial growth and macrophage proliferation. Nanoscale.

[179] Liu X., Chen D., Su J., Zheng R., Ning Z., Zhao M., Zhu B., Li Y. (2022). Selenium nanoparticles inhibited H1N1 influenza virus-induced apoptosis by ROS-mediated signaling pathways. RSC Adv..

[180] Wang C., Chen H., Chen D., Zhao M., Lin Z., Guo M., Xu T., Chen Y., Hua L., Lin T. (2020). The Inhibition of H1N1 Influenza Virus-Induced Apoptosis by Surface Decoration of Selenium Nanoparticles with β-Thujaplicin through Reactive Oxygen Species-Mediated AKT and p53 Signaling Pathways. ACS Omega.

[181] Lin Z., Li Y., Gong G., Xia Y., Wang C., Chen Y., Hua L., Zhong J., Tang Y., Liu X. (2018). Restriction of H1N1 influenza virus infection by selenium nanoparticles loaded with ribavirin via resisting caspase-3 apoptotic pathway. Int. J. Nanomed..

[182] Li Y., Lin Z., Guo M., Zhao M., Xia Y., Wang C., Xu T., Zhu B. (2018). Inhibition of H1N1 influenza virus-induced apoptosis by functionalized selenium nanoparticles with amantadine through ROS-mediated AKT signaling pathways. Int. J. Nanomed..

[183] Zhong J., Xia Y., Hua L., Liu X., Xiao M., Xu T., Zhu B., Cao H. (2019). Functionalized selenium nanoparticles enhance the anti-EV71 activity of oseltamivir in human astrocytoma cell model. Artif. Cells Nanomed. Biotechnol..

[184] Lin Z., Li Y., Xu T., Guo M., Wang C., Zhao M., Chen H., Kuang J., Li W., Zhang Y. (2020). Inhibition of Enterovirus 71 by Selenium Nanoparticles Loaded with siRNA through Bax Signaling Pathways. ACS Omega.

[185] Makhlof M.E.M., Albalwe F.M., Al-Shaikh T.M., El-Sheekh M.M. (2022). Suppression Effect of Ulva lactuca Selenium Nanoparticles (USeNPs) on HepG2 Carcinoma Cells Resulting from Degradation of Epidermal Growth Factor Receptor (EGFR) with an Evaluation of Its Antiviral and Antioxidant Activities. Appl. Sci..

[186] Touliabah H.E., El-Sheekh M.M., Makhlof M.E.M. (2022). Evaluation of *Polycladia myrica* mediated selenium nanoparticles (PoSeNPS) cytotoxicity against PC-3 cells and antiviral activity against HAV HM175 (Hepatitis A), HSV-2 (Herpes simplex II), and Adenovirus strain 2. Front. Mar. Sci..

[187] Li Y., Lin Z., Guo M., Xia Y., Zhao M., Wang C., Xu T., Chen T., Zhu B. (2017). Inhibitory activity of selenium nanoparticles functionalized with oseltamivir on H1N1 influenza virus. Int. J. Nanomed..

[188] Zhang H., Li Z., Dai C., Wang P., Fan S., Yu B., Qu Y. (2021). Antibacterial properties and mechanism of selenium nanoparticles synthesized by *Providencia* sp. DCX. Environ. Res..

[189] Mao L., Wang L., Zhang M., Ullah M., Liu L., Zhao W., Li Y., Ahmed A.A.Q., Cheng H., Shi Z. (2021). In Situ Synthesized Selenium Nanoparticles-Decorated Bacterial Cellulose/Gelatin Hydrogel with Enhanced Antibacterial, Antioxidant, and Anti-Inflammatory Capabilities for Facilitating Skin Wound Healing. Adv. Healthc. Mater..

[190] Cremonini E., Boaretti M., Vandecandelaere I., Zonaro E., Coenye T., Lleo M.M., Lampis S., Vallini G. (2018). Biogenic selenium nanoparticles synthesized by Stenotrophomonas maltophilia SeITE02 loose antibacterial and antibiofilm efficacy as a result of the progressive alteration of their organic coating layer. Microb. Biotechnol..

[191] Abbas H.S., Abou Baker D.H., Ahmed E.A. (2021). Cytotoxicity and antimicrobial efficiency of selenium nanoparticles biosynthesized by *Spirulina platensis*. Arch. Microbiol..

[192] Pekarkova J., Gablech I., Fialova T., Bilek O., Fohlerova Z. (2021). Modifications of Parylene by Microstructures and Selenium Nanoparticles: Evaluation of Bacterial and Mesenchymal Stem Cell Viability. Front. Bioeng. Biotechnol..

[193] Elakraa A., Salah Salem S., El-Sayyad G., Salah Attia M. (2022). Cefotaxime incorporated bimetallic silver-selenium nanoparticles: Promising antimicrobial synergism, antibiofilm activity, and bacterial membrane leakage reaction mechanism. RSC Adv..

[194] Galkina K.V., Zubareva V.M., Kashko N.D., Lapashina A.S., Markova O.V., Feniouk B.A., Knorre D.A. (2022). Heterogeneity of Starved Yeast Cells in IF1 Levels Suggests the Role of This Protein in vivo. Front. Microbiol..

[195] Hyrslova I., Kaňa A., Kantorova V., Krausova G., Mrvikova I., Doskocil I. (2022). Selenium accumulation and biotransformation in *Streptococcus*, *Lactococcus*, and *Enterococcus strains*. J. Funct. Foods.

[196] Tendenedzai J.T., Chirwa E.M.N., Brink H.G. (2022). *Enterococcus* spp. Cell-Free Extract: An Abiotic Route for Synthesis of Selenium Nanoparticles (SeNPs), Their Characterisation and Inhibition of *Escherichia coli*. Nanomaterials.

[197] Tran P.A., O’Brien-Simpson N., Reynolds E.C., Pantarat N., Biswas D.P., O’Connor A.J. (2016). Low cytotoxic trace element selenium nanoparticles and their differential antimicrobial properties against *S. aureus* and *E. coli*. Nanotechnology.

[198] Chandramohan S., Sundar K., Muthukumaran A. (2019). Hollow selenium nanoparticles from potato extract and investigation of its biological properties and developmental toxicity in zebrafish embryos. IET Nanobiotechnol..

[199] Huang T., Holden J.A., Heath D.E., O’Brien-Simpson N.M., O’Connor A.J. (2019). Engineering highly effective antimicrobial selenium nanoparticles through control of particle size. Nanoscale.

[200] Chung S., Zhou R., Webster T. (2020). Green Synthesized BSA-Coated Selenium Nanoparticles Inhibit Bacterial Growth While Promoting Mammalian Cell Growth. Int. J. Nanomed..

[201] Huang T., Kumari S., Herold H., Bargel H., Aigner T., Heath D., O’Brien-Simpson N., O’Connor A., Scheibel T. (2020). Enhanced Antibacterial Activity of Se Nanoparticles Upon Coating with Recombinant Spider Silk Protein eADF4(κ16). Int. J. Nanomed..

[202] El-Sayyad G.S., El-Bastawisy H.S., Gobara M., El-Batal A.I. (2020). Gentamicin-Assisted Mycogenic Selenium Nanoparticles Synthesized Under Gamma Irradiation for Robust Reluctance of Resistant Urinary Tract Infection-Causing Pathogens. Biol. Trace Elem. Res..

[203] Guisbiers G., Wang Q., Khachatryan E., Mimun L., Mendoza-Cruz R., Larese-Casanova P., Webster T., Nash K. (2016). Inhibition of *E. coli* and *S. aureus* with selenium nanoparticles synthesized by pulsed laser ablation in deionized water. Int. J. Nanomed..

[204] Shahmoradi S., Shariati A., Amini S.M., Zargar N., Yadegari Z., Darban-Sarokhalil D. (2022). The application of selenium nanoparticles for enhancing the efficacy of photodynamic inactivation of planktonic communities and the biofilm of *Streptococcus mutans*. BMC Res. Notes.

[205] Hernández-Díaz J.A., Garza-García J.J., León-Morales J.M., Zamudio-Ojeda A., Arratia-Quijada J., Velázquez-Juárez G., López-Velázquez J.C., García-Morales S. (2021). Antibacterial Activity of Biosynthesized Selenium Nanoparticles Using Extracts of Calendula officinalis against Potentially Clinical Bacterial Strains. Molecules.

[206] Mansouri-Tehrani H.A., Keyhanfar M., Behbahani M., Dini G. (2021). Synthesis and characterization of algae-coated selenium nanoparticles as a novel antibacterial agent against *Vibrio harveyi*, a *Penaeus vannamei* pathogen. Aquaculture.

[207] Bagheri-Josheghani S., Bakhshi B. (2022). Investigation of the Antibacterial and Antibiofilm Activity of Selenium Nanoparticles against *Vibrio cholerae* as a Potent Therapeutics. Can. J. Infect. Dis. Med. Microbiol..

[208] Alghuthaymi M.A. (2022). Antibacterial action of insect chitosan/gum Arabic nanocomposites encapsulating eugenol and selenium nanoparticles. J. King Saud Univ. Sci..

[209] Hosseini Bafghi M., Zarrinfar H., Darroudi M., Zargar M., Nazari R. (2022). Green synthesis of selenium nanoparticles and evaluate their effect on the expression of ERG3, ERG11 and FKS1 antifungal resistance genes in *Candida albicans* and *Candida glabrata*. Lett. Appl. Microbiol..

[210] El-Saadony M.T., Saad A.M., Taha T.F., Najjar A.A., Zabermawi N.M., Nader M.M., AbuQamar S.F., El-Tarabily K.A., Salama A. (2021). Selenium nanoparticles from *Lactobacillus paracasei* HM1 capable of antagonizing animal pathogenic fungi as a new source from human breast milk. Saudi J. Biol. Sci..

[211] Safaei M., Mozaffari H.R., Moradpoor H., Imani M.M., Sharifi R., Golshah A. (2022). Optimization of Green Synthesis of Selenium Nanoparticles and Evaluation of Their Antifungal Activity against Oral *Candida albicans* Infection. Adv. Mater. Sci. Eng..

[212] Lara H., Guisbiers G., Mendoza J., Mimun L., Vincent B., Lopez-Ribot J., Nash K. (2018). Synergistic antifungal effect of chitosan-stabilized selenium nanoparticles synthesized by pulsed laser ablation in liquids against *Candida albicans* biofilms. Int. J. Nanomed..

[213] Shahbaz M., Akram A., Raja N.I., Mukhtar T., Mehak A., Fatima N., Ajmal M., Ali K., Mustafa N., Abasi F. (2023). Antifungal activity of green synthesized selenium nanoparticles and their effect on physiological, biochemical, and antioxidant defense system of mango under mango malformation disease. PLoS ONE.

[214] Lazcano-Ramírez H.G., Garza-García J.J.O., Hernández-Díaz J.A., León-Morales J.M., Macías-Sandoval A.S., García-Morales S. (2023). Antifungal Activity of Selenium Nanoparticles Obtained by Plant-Mediated Synthesis. Antibiotics.

[215] El-Saadony M.T., Saad A.M., Najjar A.A., Alzahrani S.O., Alkhatib F.M., Shafi M.E., Selem E., Desoky E.-S.M., Fouda S.E.E., El-Tahan A.M. (2021). The use of biological selenium nanoparticles to suppress *Triticum aestivum* L. crown and root rot diseases induced by Fusarium species and improve yield under drought and heat stress. Saudi J. Biol. Sci..

[216] Bafghi M.H., Nazari R., Darroudi M., Zargar M., Zarrinfar H. (2022). The effect of biosynthesized selenium nanoparticles on the expression of CYP51A and HSP90 antifungal resistance genes in *Aspergillus fumigatus* and *Aspergillus flavus*. Biotechnol. Prog..

[217] Shahbaz M., Fatima N., Mashwani Z.-u.-R., Akram A., Haq E.U., Mehak A., Abasi F., Ajmal M., Yousaf T., Raja N.I. (2022). Effect of Phytosynthesized Selenium and Cerium Oxide Nanoparticles on Wheat (*Triticum aestivum* L.) against Stripe Rust Disease. Molecules.

[218] Salem M.F., Abd-Elraoof W.A., Tayel A.A., Alzuaibr F.M., Abonama O.M. (2022). Antifungal application of biosynthesized selenium nanoparticles with pomegranate peels and nanochitosan as edible coatings for citrus green mold protection. J. Nanobiotechnol..

[219] Hashem A.H., Abdelaziz A.M., Askar A.A., Fouda H.M., Khalil A.M.A., Abd-Elsalam K.A., Khaleil M.M. (2021). *Bacillus megaterium*-Mediated Synthesis of Selenium Nanoparticles and Their Antifungal Activity against *Rhizoctonia solani* in Faba Bean Plants. J. Fungi.

[220] Joshi S.M., De Britto S., Jogaiah S., Ito S.-I. (2019). Mycogenic Selenium Nanoparticles as Potential New Generation Broad Spectrum Antifungal Molecules. Biomolecules.

[221] Filipović N., Ušjak D., Milenković M.T., Zheng K., Liverani L., Boccaccini A.R., Stevanović M.M. (2021). Comparative Study of the Antimicrobial Activity of Selenium Nanoparticles with Different Surface Chemistry and Structure. Front. Bioeng. Biotechnol..

[222] Tuyen N.N.K., Huy V.K., Duy N.H., An H., Nam N.T.H., Dat N.M., Huong Q.T.T., Trang N.L.P., Anh N.D.P., Thy L.T.M. (2023). Green synthesis of selenium nanorods using *Muntigia calabura* leaf extract: Effect of pH on characterization and bioactivities. Res. Sq..

[223] Liu P., Ma Y., Cai W., Wang Z., Wang J., Qi L., Chen D. (2007). Photoconductivity of single-crystalline selenium nanotubes. Nanotechnology.

[224] Cheren’kaja T.V., Borisova L.A., Aleksandrova I.V., Kosolapov D.A. (2013). The etiological structure of bacteremia and sepsis causative agents in patients with intensive care in an emergency hospital. Neotlozhnaya Meditsinskaja Pomos..

[225] Lesnichaya M.V., Malysheva S.F., Belogorlova N.A., Graskova I.A., Gazizova A.V., Perfilyeva A.I., Nozhkina O.A., Sukhov B.G. (2019). Synthesis and antimicrobial activity of arabinogalactan-stabilized selenium nanoparticles from sodium bis(2-phenylethyl)diselenophosphinate. Russ. Chem. Bull..

[226] Sahoo B., Leena Panigrahi L., Jena S., Jha S., Arakha M. (2023). Oxidative stress generated due to photocatalytic activity of biosynthesized selenium nanoparticles triggers cytoplasmic leakage leading to bacterial cell death. RSC Adv..

[227] Mühling M., Bradford A., Readman J.W., Somerfield P.J., Handy R.D. (2009). An investigation into the effects of silver nanoparticles on antibiotic resistance of naturally occurring bacteria in an estuarine sediment. Mar. Environ. Res..

[228] Cremonini E., Zonaro E., Donini M., Lampis S., Boaretti M., Dusi S., Melotti P., Lleo M.M., Vallini G. (2016). Biogenic selenium nanoparticles: Characterization, antimicrobial activity and effects on human dendritic cells and fibroblasts. Microb. Biotechnol..

[229] Garg A., Singh C., Pradhan D., Ghosh G., Rath G. (2020). Topical application of nanoparticles integrated supramolecular hydrogels for the potential treatment of seborrhoeic dermatitis. Pharm. Dev. Technol..

[230] Parsamehr N., Rezaie S., Khodavaisy S., Salari S., Hadizadeh S., Kord M., Ayatollahi Mousavi S.A. (2017). Effect of biogenic selenium nanoparticles on ERG11 and CDR1 gene expression in both fluconazole-resistant and -susceptible *Candida albicans* isolates. Curr. Med. Mycol..

[231] Nagajyothi P.C., Sreekanth T.V.M., Tettey C.O., Jun Y.I., Mook S.H. (2014). Characterization, antibacterial, antioxidant, and cytotoxic activities of ZnO nanoparticles using Coptidis Rhizoma. Bioorganic Med. Chem. Lett..

[232] Rao K., Imran M., Jabri T., Ali I., Perveen S., Shafiullah, Ahmed S., Shah M.R. (2017). Gum tragacanth stabilized green gold nanoparticles as cargos for Naringin loading: A morphological investigation through AFM. Carbohydr. Polym..

[233] Song Z., Hrbek J., Osgood R. (2005). Formation of TiO_2_ Nanoparticles by Reactive-Layer-Assisted Deposition and Characterization by XPS and STM. Nano Lett..

[234] Das B., Dash S.K., Mandal D., Ghosh T., Chattopadhyay S., Tripathy S., Das S., Dey S.K., Das D., Roy S. (2017). Green synthesized silver nanoparticles destroy multidrug resistant bacteria via reactive oxygen species mediated membrane damage. Arab. J. Chem..

[235] Bell N.C., Minelli C., Tompkins J., Stevens M.M., Shard A.G. (2012). Emerging Techniques for Submicrometer Particle Sizing Applied to Stöber Silica. Langmuir.

[236] Ehara K., Sakurai H. (2010). Metrology of airborne and liquid-borne nanoparticles: Current status and future needs. Metrologia.

[237] Henriquez R.R., Ito T., Sun L., Crooks R.M. (2004). The resurgence of Coulter counting for analyzing nanoscale objects. Analyst.

[238] Neville F., Broderick M.J.F., Gibson T., Millner P.A. (2011). Fabrication and Activity of Silicate Nanoparticles and Nanosilicate-Entrapped Enzymes Using Polyethyleneimine As a Biomimetic Polymer. Langmuir.

[239] Gudkov S.V., Astashev M.E., Baimler I.V., Uvarov O.V., Voronov V.V., Simakin A.V. (2022). Laser-Induced Optical Breakdown of an Aqueous Colloidal Solution Containing Terbium Nanoparticles: The Effect of Oxidation of Nanoparticles. J. Phys. Chem. B.

[240] Singh V., Shrivastava A., Wahi N. (2015). Biosynthesis of silver nanoparticles by plants crude extracts and their characterization using UV, XRD, TEM and EDX. Afr. J. Biotechnol..

[241] Naraginti S., Li Y. (2017). Preliminary investigation of catalytic, antioxidant, anticancer and bactericidal activity of green synthesized silver and gold nanoparticles using *Actinidia deliciosa*. J. Photochem. Photobiol. B Biol..

[242] Zad Z.R., Davarani S.S.H., Taheri A., Bide Y. (2018). A yolk shell Fe_3_O_4_@PA-Ni@Pd/Chitosan nanocomposite -modified carbon ionic liquid electrode as a new sensor for the sensitive determination of fluconazole in pharmaceutical preparations and biological fluids. J. Mol. Liq..

[243] Mittal A.K., Chisti Y., Banerjee U.C. (2013). Synthesis of metallic nanoparticles using plant extracts. Biotechnol. Adv..

[244] Wang T., Lin J., Chen Z., Megharaj M., Naidu R. (2014). Green synthesized iron nanoparticles by green tea and eucalyptus leaves extracts used for removal of nitrate in aqueous solution. J. Clean. Prod..

[245] Sana S.S., Dogiparthi L.K. (2018). Green synthesis of silver nanoparticles using *Givotia moluccana* leaf extract and evaluation of their antimicrobial activity. Mater. Lett..

[246] Venkateswarlu S., Natesh Kumar B., Prasad C.H., Venkateswarlu P., Jyothi N.V.V. (2014). Bio-inspired green synthesis of Fe_3_O_4_ spherical magnetic nanoparticles using *Syzygium cumini* seed extract. Phys. B Condens. Matter.

[247] Burmistrov D.E., Serov D.A., Simakin A.V., Baimler I.V., Uvarov O.V., Gudkov S.V. (2022). A Polytetrafluoroethylene (PTFE) and Nano-Al_2_O_3_ Based Composite Coating with a Bacteriostatic Effect against *E. coli* and Low Cytotoxicity. Polymers.

[248] Bai K., Hong B., Huang W., He J. (2020). Selenium-Nanoparticles-Loaded Chitosan/Chitooligosaccharide Microparticles and Their Antioxidant Potential: A Chemical and In Vivo Investigation. Pharmaceutics.

[249] Shurygina I.A., Trukhan I.S., Dremina N.N., Shurygin M.G. (2021). Selenium Nanoparticles. Nanotechnology in Medicine.

[250] Kumar C.M.V., Karthick V., Inbakandan D., Kumar V.G., Rene E.R., Dhas T.S., Ravi M., Sowmiya P., Das C.G.A. (2022). Effect of selenium nanoparticles induced toxicity on the marine diatom *Chaetoceros gracilis*. Process Saf. Environ. Prot..

[251] Crisan M.C., Teodora M., Lucian M. (2021). Copper Nanoparticles: Synthesis and Characterization, Physiology, Toxicity and Antimicrobial Applications. Appl. Sci..

[252] Ruparelia J.P., Chatterjee A.K., Duttagupta S.P., Mukherji S. (2008). Strain specificity in antimicrobial activity of silver and copper nanoparticles. Acta Biomater..

[253] Hou J., Wang X., Hayat T., Wang X. (2017). Ecotoxicological effects and mechanism of CuO nanoparticles to individual organisms. Environ. Pollut..

[254] Tang H., Xu M., Luo J., Zhao L., Ye G., Shi F., Lv C., Chen H., Wang Y., Li Y. (2019). Liver toxicity assessments in rats following sub-chronic oral exposure to copper nanoparticles. Environ. Sci. Eur..

[255] Ferro C., Florindo H.F., Santos H.A. (2021). Selenium Nanoparticles for Biomedical Applications: From Development and Characterization to Therapeutics. Adv. Healthc. Mater..

[256] Ramamurthy C.H., Sampath K.S., Arunkumar P., Kumar M.S., Sujatha V., Premkumar K., Thirunavukkarasu C. (2013). Green synthesis and characterization of selenium nanoparticles and its augmented cytotoxicity with doxorubicin on cancer cells. Bioprocess Biosyst. Eng..

[257] Bhattacharjee A., Basu A., Biswas J., Sen T., Bhattacharya S. (2016). Chemoprotective and chemosensitizing properties of selenium nanoparticle (Nano-Se) during adjuvant therapy with cyclophosphamide in tumor-bearing mice. Mol. Cell. Biochem..

[258] Zambonino M.C., Quizhpe E.M., Mouheb L., Rahman A., Agathos S.N., Dahoumane S.A. (2023). Biogenic Selenium Nanoparticles in Biomedical Sciences: Properties, Current Trends, Novel Opportunities and Emerging Challenges in Theranostic Nanomedicine. Nanomaterials.

[259] Liu W., Li X., Wong Y.-S., Zheng W., Zhang Y., Cao W., Chen T. (2012). Selenium Nanoparticles as a Carrier of 5-Fluorouracil to Achieve Anticancer Synergism. ACS Nano.

[260] Prasad K.S., Selvaraj K. (2014). Biogenic Synthesis of Selenium Nanoparticles and Their Effect on As(III)-Induced Toxicity on Human Lymphocytes. Biol. Trace Elem. Res..

[261] Qiu Y., Chen X., Chen Z., Zeng X., Yue T., Yuan Y. (2022). Effects of Selenium Nanoparticles on Preventing Patulin-Induced Liver, Kidney and Gastrointestinal Damage. Foods.

[262] Deng W., Xie Q., Wang H., Ma Z., Wu B., Zhang X. (2017). Selenium nanoparticles as versatile carriers for oral delivery of insulin: Insight into the synergic antidiabetic effect and mechanism. Nanomed. Nanotechnol. Biol. Med..

[263] Liu Y., Zeng S., Liu Y., Wu W., Shen Y., Zhang L., Li C., Chen H., Liu A., Shen L. (2018). Synthesis and antidiabetic activity of selenium nanoparticles in the presence of polysaccharides from *Catathelasma ventricosum*. Int. J. Biol. Macromol..

[264] Abdel Moneim A., Al-Quraishy S., Dkhil M.A. (2015). Anti-hyperglycemic activity of selenium nanoparticles in streptozotocin-induced diabetic rats. Int. J. Nanomed..

[265] Yang L., Wang W., Chen J., Wang N., Zheng G. (2018). A comparative study of resveratrol and resveratrol-functional selenium nanoparticles: Inhibiting amyloid β aggregation and reactive oxygen species formation properties. J. Biomed. Mater. Res. Part A.

[266] Mahmoudvand H., Shakibaie M., Tavakoli R., Jahanbakhsh S., Sharifi I. (2014). In Vitro Study of Leishmanicidal Activity of Biogenic Selenium Nanoparticles against Iranian Isolate of Sensitive and Glucantime-Resistant *Leishmania tropica*. Iran. J. Parasitol..

[267] Wlodkowic D., Telford W., Skommer J., Darzynkiewicz Z. (2011). Apoptosis and Beyond: Cytometry in Studies of Programmed Cell Death. Methods Cell Biol..

[268] Malekifard F., Tavassoli M., Vaziri K. (2020). In Vitro Assessment Antiparasitic Effect of Selenium and Copper Nanoparticles on *Giardia deodenalis* Cyst. Iran. J. Parasitol..

[269] Arafa F.M., Mogahed N.M.F.H., Eltarahony M.M., Diab R.G. (2023). Biogenic selenium nanoparticles: Trace element with promising anti-toxoplasma effect. Pathog. Glob. Health.

[270] Mahmoudvand H., Fasihi Harandi M., Shakibaie M., Aflatoonian M.R., ZiaAli N., Makki M.S., Jahanbakhsh S. (2014). Scolicidal effects of biogenic selenium nanoparticles against protoscolices of hydatid cysts. Int. J. Surg..

[271] Mohammed S., Ali A.A. (2022). Effect of selenium nanoparticles against protoscoleces of *Echinococcus granulosus* in vitro and hydatid cysts in mice. Iraqi J. Vet. Sci..

[272] Sarhan M.H., Farghaly A., Abd El-Aal N.F., Mohammed Farag S., Ahmed Ali A., Farag T.I. (2022). Egyptian propolis and selenium nanoparticles against murine trichinosis: A novel therapeutic insight. J. Helminthol..

